# Engineered extracellular vesicles-like biomimetic nanoparticles as an emerging platform for targeted cancer therapy

**DOI:** 10.1186/s12951-023-02064-1

**Published:** 2023-08-22

**Authors:** Xinyi Liu, Chunxiu Xiao, Kai Xiao

**Affiliations:** 1 Precision Medicine Research Center, Sichuan Provincial Key Laboratory of Precision Medicine, Frontiers Science Center for Disease-Related Molecular Network, National Clinical Research Center for Geriatrics, West China Hospital, Sichuan University, Chengdu, 610041 China; 2 Tianfu Jingcheng Laboratory (Frontier Medical Center), Chengdu, 610041 China

**Keywords:** Extracellular vesicles (EVs), Biomimetic nanoparticles, Drug delivery system, Engineering strategy, Functionalization, Targeted cancer therapy

## Abstract

Nanotechnology offers the possibility of revolutionizing cancer theranostics in the new era of precision oncology. Extracellular vesicles (EVs)-like biomimetic nanoparticles (EBPs) have recently emerged as a promising platform for targeted cancer drug delivery. Compared with conventional synthetic vehicles, EBPs have several advantages, such as lower immunogenicity, longer circulation time, and better targeting capability. Studies on EBPs as cancer therapeutics are rapidly progressing from in vitro experiments to in vivo animal models and early-stage clinical trials. Here, we describe engineering strategies to further improve EBPs as effective anticancer drug carriers, including genetic manipulation of original cells, fusion with synthetic nanomaterials, and direct modification of EVs. These engineering approaches can improve the anticancer performance of EBPs, especially in terms of tumor targeting effectiveness, stealth property, drug loading capacity, and integration with other therapeutic modalities. Finally, the current obstacles and future perspectives of engineered EBPs as the next-generation delivery platform for anticancer drugs are discussed.

## Introduction

Cancer poses a serious threat to human health [1]. Chemotherapeutic drugs, one of the principal means of cancer treatment, lack selectivity for cancer cells, leading to toxic side effects such as bone marrow suppression and gastrointestinal issues, as well as the emergence of drug resistance [2]. Targeted drug delivery is an important research direction in the field of precision oncology, which can effectively improve the efficacy of anticancer drugs and reduce their toxicity. Nanoparticle-based drug delivery systems possess some essential features, such as high drug loading capacity, long circulation time, and high bioavailability, which can increase drug concentration in specific organs or tissues, ultimately improving treatment effectiveness and avoiding side effects [3]. Over the past decade, organic and inorganic materials such as polymers, liposomes, and proteins have been developed as nanocarriers to improve the delivery efficiency of therapeutic molecules or genes [4]. However, it is worth noting that after systemic administration, only 0.7% of the injected dose of nanoparticles reaches the tumor site [5]. In addition, recent clinical data indicate that conventional nanoparticles are not very effective in terms of drug specificity and effectiveness, and the incidence of side effects is high. Therefore, novel nanocarriers need to be developed to further improve tumor enrichment of anticancer drugs, thus enhancing their therapeutic efficacy with low toxicity [6].

Extracellular vesicles (EVs) [7], a new class of drug nanocarriers, have gained increasing attention in recent years. According to the definition of the International Society for Extracellular Vesicles (ISEV), EVs are naturally released particles from cells, including exosomes, microvesicles, microparticles, apoptotic bodies, and other non-replicating extracellular vesicle subgroups. Studies have shown that EVs are important mediators of intercellular communication and contain diverse bioactive substances like nucleic acids, proteins, lipids, and metabolites [8–10]. These substances play a vital role in physiological and pathological processes by transmitting information through autocrine, paracrine, or endocrine pathways to specific cells. EVs have potential as diagnostic and therapeutic tools for disease detection, prevention, immune system regulation, antitumor therapy, and wound healing [11]. In 2007, Valadi et al. demonstrated that mast cell-derived EVs can deliver functional mRNA [12]. Since then, EVs have been used to transport various therapeutics including siRNAs, miRNAs, small molecule drugs, and proteins. EVs can be derived from various cell lines, primary cells, human plasma, fetal bovine serum, urine, milk, and edible plants, which provides a certain degree of biocompatibility and the ability to evade the immune system [13–17]. In addition, various types of cells are capable of offering diverse biological functions to EVs. However, the clinical application of this approach is constrained by factors including low yield, low cost-effectiveness, insufficient targeting ability, limited drug loading capacity, and inadequate drug release capability. Current isolation methods can only produce a small number of EVs in a short period of time, and inadequate separation techniques can introduce protein contaminants in the final product. This contamination presents challenges in differentiating between EVs and non-EVs for downstream analysis. Furthermore, unmodified EVs may face obstacles in targeting, which may potentially limit their ability to reach target tissues. Additionally, due to the absence of standardized preparation schemes, EVs obtained through different strategies possess varying properties and functions, making large-scale production and clinical application challenging.

Therefore, scientists have attempted to address the aforementioned limitations of EVs through various engineering approaches, including internal loading and external modification, fusion with other membrane components or various types of nanoparticles, and even the disruption of cell membrane structure and recombination of EVs analogues. These unnaturally engineered nanoparticles have undergone a series of manual operations and are no longer suitable for the definition of EVs. Therefore, it is necessary to define such EVs-based nanoparticles, which will facilitate subsequent analysis, research, and the formulation of corresponding standards and specifications. Based on the fact that these engineered nanoparticles have vesicle-like structures similar to EVs as well as biomimetic properties that mimic cell membranes, we refer to them as EV-like biomimetic nanoparticles (EBPs). All artificially engineered nanoparticles based on EVs or EV analogues, as well as those reconstituted with cell membrane components, are classified as EBPs. Table 1 presents the description and comparison of EVs, EBPs, and other non-biomimetic synthetic nanomaterials such as commonly used liposomes and polymers.

**Table 1:** Comparison of EVs, EBPs and non-biomimetic synthetic nanoparticles

	EVs	Non-biomimetic synthetic nanoparticles	EBPs
Generation approaches	Cellular secretion	Synthetic techniques	Combination of both
Structure	Nanovesicles composed of cell membranes	Multiple types	Cell membrane vesicles or nanoparticles mixed with cell membrane components
Functionality	Limited bioactive functions	Multi-functional, easy to implement physical and chemical modifications	Multi-functional, easy to implement physical, chemical and biological modifications
Biosafety and immunogenicity	Good biocompatibility, non-human cells may be immunogenic	The synthetic material itself may have certain toxicity and immunogenicity	Good biocompatibility, adjustable immunogenicity
Productivity	Low	High	Hopefully improved
Drug carrying capacity	Difficult to load hydrophilic drugs	High	High
Stability	High	Not always	High
Clinical research	Less, the actual application effect is unknown	More	Less
Application prospect	Poor repeatability	Simple, standard production and loading methods	Expected to have more scalable production approaches

EBPs can be invoked as a substitute for EVs to exert their biological function, with simpler preparation methods, higher yield and production efficiency, and more effective combination therapy integration, showing better application prospects. Compared with traditional methods, artificial methods can significantly increase the yield and shorten the preparation time of EBPs [18]. The most common artificial preparation method for EBPs is to extrude vesicles through a membrane filter [19, 20]. When combined with other nanomaterials, the membrane vesicles can be squeezed again to surround the particles. The EBPs prepared by wrapping organic or inorganic synthesized nanoparticles in a cell membrane not only have certain characteristics of synthesized nanoparticles, but also have the natural or modified properties of the source cell [21]. In addition, the combination with other nanocarriers with higher drug loading capacities, such as liposomes and polymeric nanoparticles, also provides a feasible option for more effective drug loading of EBPs. Carboxyfluorescein succinyl ester (CFSE), calcein acetoxymethyl (calcein-AM), and the fluorescent dyes PKH and DiI/O/D/R have been widely used to label EBPs [22–24]. Nevertheless, these fluorescent dyes may lead to false positive signals arising from dye-induced micelles, and the resulting fluorescent signal may persist in the tissue beyond the duration of the EBPs themselves. To circumvent the issue of false positive signals, it may be advisable to consider alternative labeling strategies such as merging EBPs with imaging-capable nanomaterials or creating fluorescent membrane proteins through genetic engineering techniques.

**Fig. 1:**
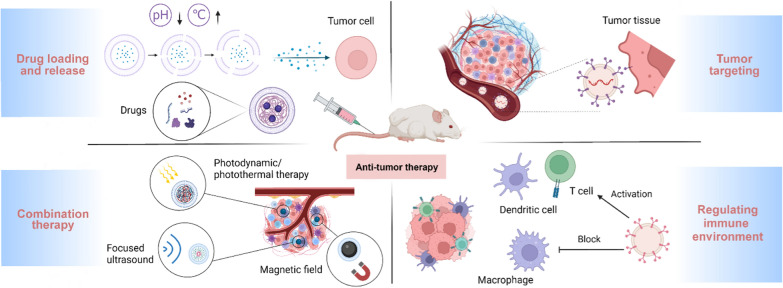
Schematic summary of engineered EBPs in targeted cancer therapy

This review aims to summarize the utilization of EBPs as drug delivery vehicles in cancer treatment through different engineering strategies, including biological, physical, and chemical approaches, and discuss the challenges, potential solutions, and future prospects of EBPs in clinical applications. Figure 1 depicts the potential applications of EBPs in the field of targeted cancer therapy, including targeting tumor tissue, improving the tumor immune microenvironment, aiding photothermal and magnetic field therapy, and enhancing drug utilization for safer and more effective treatment. For instance, EBPs can be surface modified with targeted molecules such as antibodies or peptides to recognize and bind specifically to receptors or antigens of tumor cells. This precise targeting facilitates the delivery of therapeutic drugs to tumor tissue, minimizing harm to normal tissues. Additionally, mixing EBPs with synthetic nanoparticles can improve the solubility, stability, and utilization of drugs. The tumor immune microenvironment can suppress the immune response, allowing tumors to evade immune surveillance. EBPs can improve the tumor microenvironment by carrying immunomodulatory molecules or specific cell types, such as dendritic cells or T cells, augmenting the efficiency of immunotherapy. Furthermore, EBPs can combine drug delivery with photothermal and magnetic field therapy to enhance antitumor efficacy. For example, EBPs enable photothermal therapy by carrying photosensitive molecules or thermal agents that, when activated, release heat or generate active substances to destroy tumor cells. Similarly, EBPs carrying magnetic nanoparticles can locate tumor sites under magnetic fields, enabling targeted therapy.

## Engineering strategies of EBPs

To improve their scalability and therapeutic outcomes, a range of engineering strategies have been implemented to construct EBPs. The utilization of these strategies endow EBPs with multifunctionality and enhanced antitumor ability. Firstly, through genetic engineering of original cells, it becomes possible to alter the surface properties and confer specific targeting capabilities of EBPs, enabling their precise delivery to the site of the disease. Secondly, the fusion of EVs or cell membrane structures with synthetic nanoparticles can improve the drug loading capacity of the obtained EBPs, enhance their stability and biocompatibility, and thus improve their therapeutic effect. Thirdly, direct modification of EVs can also alter the intracellular delivery mechanism of nanoparticles, further augmenting their therapeutic impact. The integration of these engineering strategies not only expands the application scope of EBPs but also paves the way for the realization of precision medicine.

### Genetic engineering of original cells

**Fig. 2:**
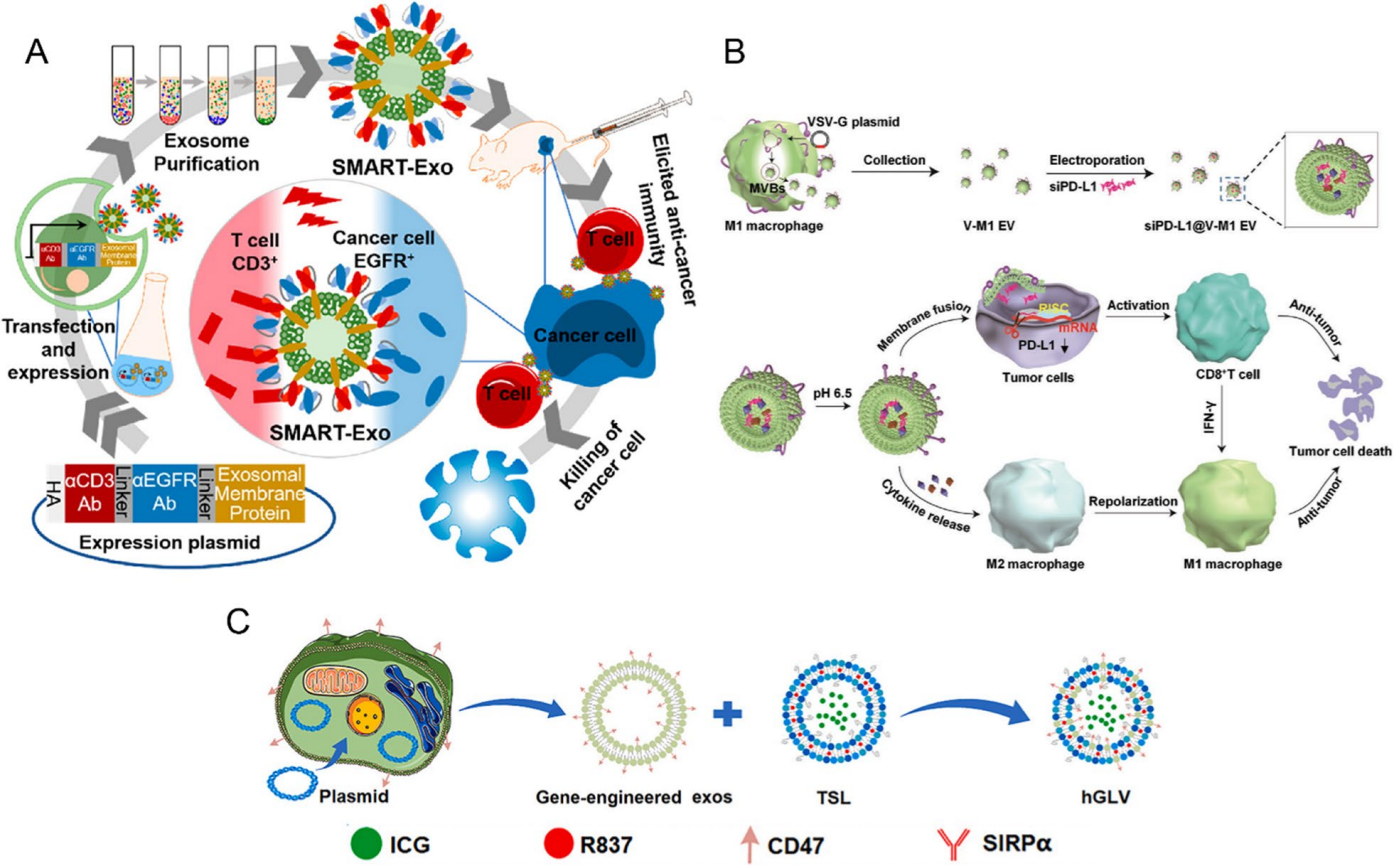
Schematic illustration of the genetically engineered EBPs. **A** Schematic diagram of the design and generation of αCD3/αEGFR synthetic multivalent antibody-displaying exosomes (SMART-Exos). It was demonstrated that the SMART-Exos, which was designed to simultaneously target CD3 and EGFR, could effectively bind to both T cells and TNBC cells expressing EGFR [25]. Reproduced with permission. Copyright 2018, American Chemical Society. **B** Schematic illustration of the virus-derived fusogenic protein VSV-G on the surface of M1 EVs loaded with siPD-L1. siRNA@V-M1 EVs can target tumors due to the native targeting capability of M1 EVs. The pH-responsive VSV-G changes to an unfolded fusion state in the acidic tumor microenvironment [26]. Reproduced with permission. Copyright 2020, Wiley. **C** The design principle of hGLV involves utilizing photothermal therapy (PTT) in combination with immunotherapy to combat tumors [27]. Copyright 2021, Elsevier. *TNBC* triple-negative breast cancer, *EGFR* epidermal growth factor receptor, *exos* exosomes, *TSL* thermosensitive liposomes, *hGLV* gene-engineered exosome-thermosensitive liposome hybrid nanovesicles, *DCs* dendritic cells, *SMART-Exos* synthetic multivalent antibody-retargeted exosomes, *VSV-G* vesicular stomatitis virus glycoprotein, *M1 EVs* M1 macrophage-derived EVs

Genetic engineering is one of the ways to obtain ideal EBPs. Genetic engineering techniques in EBPs display functional ligands on the membrane of donor cells by constructing plasmids and overexpressing proteins. Figure 2 illustrates three examples of genetic engineering modifications. Cheng et al. decorated two distinct antibodies on the surface of the EBPs, one targeting CD3 on T cells and the other targeting epidermal growth factor receptor (EGFR) on triple negative breast cancer (TNBC) cells [25]. Both in vitro and in vivo experiments have demonstrated that the modified SMART-EBPs can induce the specific killing of immune cells against tumors. This suggests that the decoration of EBPs with antibodies can enhance their effectiveness in targeting and eliminating cancer cells. In Fig. 2B, vesicular stomatitis virus glycoprotein (VSV-G) was introduced onto the surface of EVs derived from M1 macrophages and loaded with siPD-L1 [26]. Following administration, EVs can be directed towards tumors through the inherent targeting ability of M1-EVs. In the acidic tumor microenvironment, the pH-responsive VSV-G may undergo a conformational change, resulting in the unfolding of the protein. This, in turn, triggered the fusion of EVs with the cell membrane, enabling them to bypass the intracellular uptake pathway. Therefore, siPD-L1 can be delivered directly into the cytoplasm, triggering the silencing of the PD-L1 gene. At the same time, M1-EVs were found to be effective in repolarizing M2-type tumor-associated macrophages (TAMs) into M1-type macrophages. The combination of siPD-L1 and M1-EVs resulted in a synergistic effect, leading to a potent anticancer immunotherapeutic effect. Cheng et al. designed a hybrid therapeutic nanovesicle that fuses genetically engineered EBPs with heat-sensitive liposomes (TSLs) for cancer therapy in combination with PTT and immunotherapy [27]. First, CT26 cells were transfected to obtain EBPs overexpressing CD47, achieving homologous cell targeting and a long cycling time in vivo. Subsequently, they were fused with TSL to form hybrid nanovesicle hGLVs, which were then loaded with the photothermal agent ICG and immune adjuvant R837 to provide photothermal effects and cytotoxicity. The combination of these technologies may provide enhanced targeting, photothermal effects, and immune stimulation for improved therapeutic outcomes.

Genetic engineering approach requires a solid understanding of the physiological conformation of membrane proteins, since the function of many membrane proteins is associated with their complex tertiary structure. In addition to functional proteins, genetic engineering techniques can also introduce noncoding RNA sequences into EBPs, such as miRNA or small interfering RNA (siRNA) [28]. Clustered Regularly Interspaced Short Palindromic Repeats (CRISPR) and its associated protein, Cas9, have emerged as powerful tools for genome editing. By using CRISPR/Cas9, researchers can target specific genomic regions and edit genetic material with high precision and accuracy [29]. CRISPR/Cas9 technology can be used to genetically edit the target protein of original cells to obtain the corresponding engineered EBPs. EBPs can also serve as functional vectors to carry CRISPR-Cas9 for gene editing purposes, leveraging their natural targeting properties and ability to penetrate cell membranes to deliver genetic material with high specificity and efficiency [30]. Overall, this genetic engineering method is easy to operate, but has the limitations of poor specificity and low loading efficiency, and cannot be used to obtain non-genetically coded products. Most experiments involving genetic modifications are designed to improve the ability of EBPs to target tumors and their surrounding sites (Table 2). Engineered EBPs have proven to have useful clinical application potential in various fields, such as cancer, tissue regeneration and repair, cardiovascular and neurological diseases [31–33], and all of them generally show better targeting and therapeutic effects than natural EVs. The genetic manipulation approach has brought more standardized products, but also faces some challenges, such as mass production, stability control, storage methods, and quality control. The bioengineering strategy is time-consuming and requires repeating the whole process each time for a new molecule. In addition, due to changes in the bioactivity of EBPs caused by genetic manipulation, it is difficult to control the density and glycosylation state of target proteins, which poses potential safety risks. Researchers can explore ways to optimize and streamline the bioengineering process to reduce the time required for each cycle. This could involve improving cloning techniques, employing automation, or developing more efficient methods for genetic manipulation. In addition, researchers need to monitor and analyze any alterations in bioactivity, immunogenicity, or potential off-target effects resulting from genetic modifications. Close attention to safety concerns and robust experimental validation can help mitigate potential risks.

**Table 2:** Genetically engineered EBPs

Sources	Membrane modification	Cargoes	Loading methods	Therapeutic applications		Refs.
HEK293T	CD47 overexpression	Erastin (Er) and Rose Bengal (RB)	Ultrasonic + electroporation	Avoided vesicles by macrophages phagocytosis, enhance blood circulation ability	Combined with iron death inducer and photosensitizer	[34]
CT26	CD47 overexpression	ICG and R837 were loaded with heat-sensitive liposomes	Freeze thawing		Photothermal therapy combined with immunoadjuvant	[27]
3T3	CD47 overexpression	Docetaxel + GM-CSF	Freeze thawing		In combination with chemotherapy	[35]
4T1	Urokinase-type plasminogen activator (uPA) overexpression	PLGA nano-carrier loaded with anti-miRNA-21/antimiRNA-10b	Extrusion	Targeted receptors specifically expressed by tumor cells and binding to RNA for anti-tumor therapy		[36]
cbMSC-hTERT	Anti-GPC3 antibody	miR-26a mimic	Electroporation	miR-26a was effectively delivered to GPC3 expressing hepatocellular carcinoma (HCC) cells and inhibited tumor proliferation		[37]
Expi293	Anti-CD3 + anti-HER2 antibody			Targeted tumor cells with high HER2 expression	As a bridge between T cells and HER2 positive tumor cells	[38]
HEK293T	Anti-HER2 antibody	miR-HER2	Transfection		Knocked down HER2 expression	[39]
HEK293T	Anti-HER2 antibody	HChrR6 mRNA	Transfection		HChrR6 converted prodrugs into cytotoxic drugs after being transported to tumor cells	[40]
HEK293T	EGF or HER2 affibody	photosensitizer (ICG)/DOX	Ultrasonic		Photodynamic therapy or in combination with chemotherapy drugs	[41]
MSCs	LAMP2b-DARPin chimeric protein (anti-HER2 antibody)	DOX	Electroporation		Combined with chemotherapy drugs to kill tumors	[42]
HEK293T	Anti-HER2 antibody	MiR-21i + 5-FU	Electroporation		Reversed drug resistance in the tumor	[43]
HEK293T	Anti-CD19-CAR	CRISPR/Cas9	Electroporation	Accumulated in tumors and effectively released the CRISPR/Cas9 system targeting MYC oncogenes in vivo and in vitro		[30]
U937	Anti-PSMA peptide	Pro-DOX agents activated by PSA	Extrusion	Targeted the tumor tissue and converted the drug to an active state at the tumor site		[44]
NSC	CXCR4 overexpression	Anti-miRNA-21/miRNA-100	Microfluidics technology	Therapeutic RNA was loaded using microfluidic technology and targeted CXCR4 ligand with high expression in tumor cells		[45]
MSCs	CXCR4 overexpression	si-Survivin	Electroporation	Improved tumor targeting and carried siRNA to suppress overexpressed Survivin gene in tumors		[46]
HEK293T	T7 overexpression	Anti-mir-221 oligonucleotides (AMOs)	Electroporation	Binded tumor cells by transferrin inhibited upregulated miRNA in human brain tumor glioblastoma		[47]
cbMSCs	iRGD peptide	Anti-mir-221 oligonucleotides (AMOs)	Electroporation	Significantly enriched in the tumor site and had a good anti-tumor effect		[48]
HUVECs, HEK293T	TRAIL overexpression	Oxaliplatin (OXA) + hydroxychloroquine (HCQ)	Mixed with PLGA nanoparticles + extrusion	Targeted the tumor and induced tumor cell apoptosis, combined with autophagy drugs and autophagy inhibitors to kill the tumor		[49]
Raw264.7	TRAIL overexpression	Triptolide (TPL)	Ultrasonic	Targeted the tumor and induced apoptosis, combined with chemotherapy drugs		[50]
MSCs	Cell penetrating peptide (CPP) and TNF-α overexpression			Anchored TNF-α to cell membranes, enhanced tumor targeting, and inhibited tumor growth under an external magnetic field		[51]
MDA-MB-231	PD-1 overexpression	PARP inhibitor	Ultrasonic	Effectively prevented PD-L1 mediated T cell inhibition and provided powerful antitumor activity		[52]
DC2.4 + DC from human	αPD1 + MHC-I + B7 overexpression			Activated both natural and depleted T cells, significantly improved antigen delivery to lymphatic organs, and produced a broad-spectrum T cell response to eliminate established tumors		[53]
Expi293F	Anti-CD3 antibody + EGFR overexpression			Induced the cross-linking of T cells to EGFR-expressing breast cancer cells, and induced an effective antitumor immune response		[25]
*E. coli* DH5α	Human papillomavirus type 16 E7 protein overexpression			Induced an E7 antigen-specific cellular immune response and inhibited tumor growth		[54]
*E. coli* DH5α	BFGF overexpression			Persistent anti-BFGF autoantibodies were induced, which inhibited tumor angiogenesis and tumor growth		[55]
MDA-MB-231	α-lactalbumin (α-LA) overexpression	Human neutrophil elastase (ELANE) + Hiltonol (TLR3 agonist)	Electroporation	Promoted the activation of cDC1s and showed strong antitumor activity		[56]
HEK293T	Hyaluronidase (PH20) overexpression	DOX	Electroporation	The immunomicroenvironment was changed from an immunosuppressive phenotype to an immunosupportive phenotype, which enhanced the delivery of chemotherapy drugs and improved the efficiency of tumor treatment		[57]
HEK293T	Prostaglandin F2 receptor negative regulator overexpression	Cholesterol conjugated ASOs STAT6	Incubation	Targeted tumor-associated macrophages and induced tumor microenvironment remodeling and CD8 T cell-mediated adaptive immune response		[58]
RAW 264.7	Vesicular stomatitis virus glycoprotein overexpression	Mouse si-PD-L1	Electroporation	The PD-L1/PD-1 pathway was blocked, and T cell recognition and M1 macrophage repolarization were reconstructed		[26]
A549		miRNA-449a		Carried the target RNAi through transcriptional transactivator protein (TAT) and the trans-activating response (TAR)		[59]

### Fusion of cell membrane nanostructures with synthetic materials

**Fig. 3:**
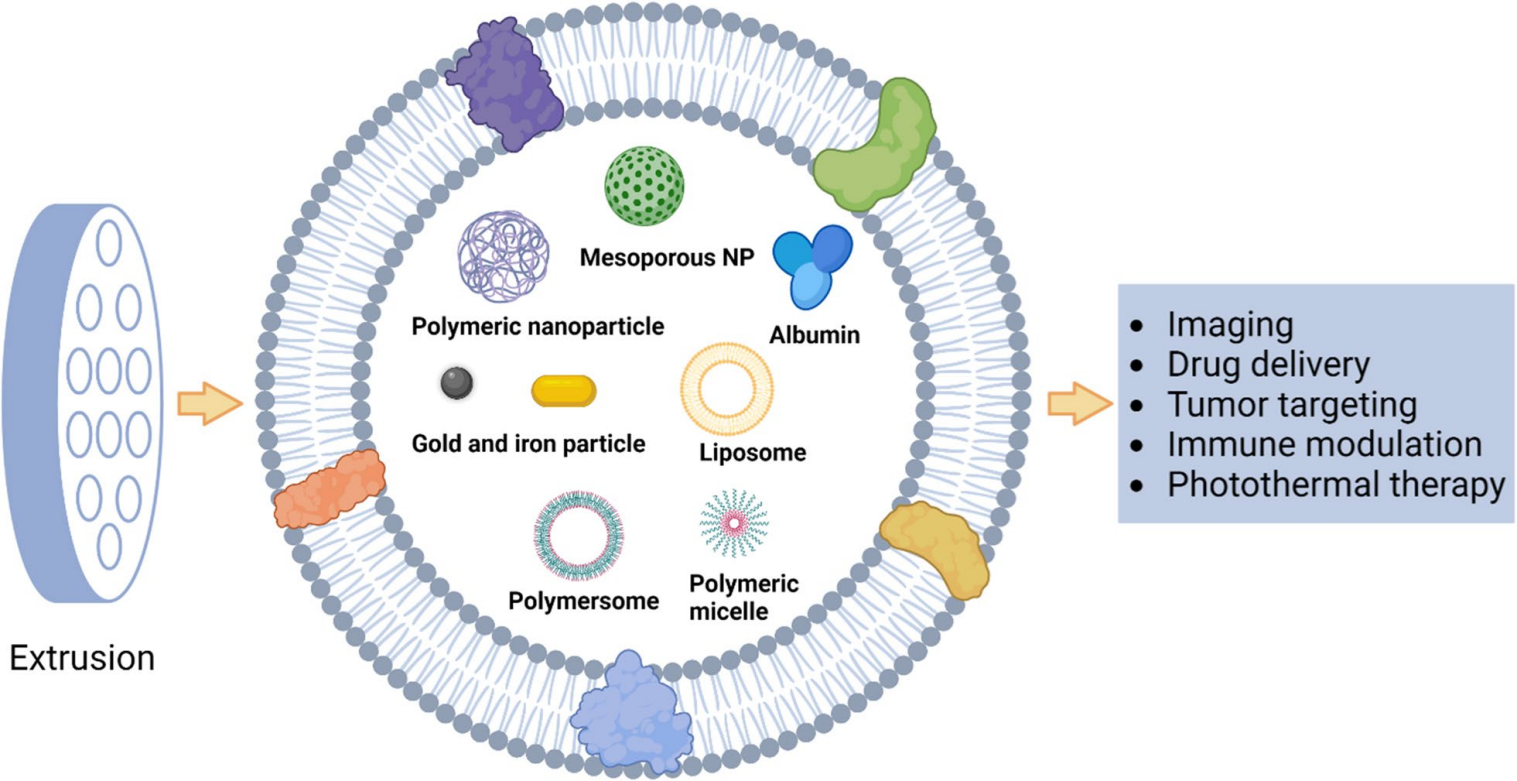
Hybrid EBPs that integrate cell membrane nanostructures with synthetic materials. Synthetic nanoparticles can combine with the plasma membrane to create membrane-coated EBPs. These hybrid EBPs not only have the unique characteristics of synthetic nanoparticles but also exhibit the inherent or adapted traits of the parent cells

Due to the properties of foreign substances, conventional nanoparticles are rapidly eliminated by the immune system after entering the bloodstream. To achieve superior anticancer effects, an ideal drug nanocarrier should have a long blood circulation time, as well as specific and efficient enrichment in tumor tissue [60]. Using the cytoplasmic membrane as the outer membrane of nanoparticles can reduce the body’s specific rejection to some extent. The cytoplasmic membrane can be obtained from cells through hypertonic treatment or by repeated freeze–thaw processes. Nanocarriers such as liposomes, polymeric micelles, and inorganic nanoparticles can all become the contents of cell membrane-coated EBPs, as shown in Fig. 3. Traditional artificial attachment of functional proteins onto nanocarriers may result in loss of the protein's natural configuration, while directly modified biofilm can avoid damage to the protein's structure and function. The application of cell membrane-coated EBPs can combine the unique properties of original cells and synthetic nanoparticles, thus achieving more effective targeting and intervention effects on cancer cells. It is worth noting that different types of nanomaterial assemblies offer various functions and properties for tumor targeting, in vivo imaging, magnetothermotherapy, photothermotherapy, and drug delivery. In addition, membrane-derived cells can be modified using genetic engineering methods to further enhance the function of cell membranes. Recent studies have demonstrated the potential of membrane-coated EBPs in cancer treatment, and a summary of their applications is provided in Table 3.

**Table 3:** Hybrid EBPs through fusing cell membrane nanostructures with synthetic materials

Type	Sources	Cargoes	Nanocarriers	Loading methods	Modification	Therapeutic applications	Refs.
Magnetic nanoparticle	4T1	Cy5-anti-miR-21	Gold-iron oxide nanoparticles (GIONs)	Extrusion		Had biomimetic properties, could be used for internal magnetic resonance imaging and produced effective photothermal effects	[71]
	Mouse serum	DOX	Raspberry-like iron oxide particle (RB)	Ultrasonic		Induced lymphocyte infiltration and inhibited tumor metastasis	[72]
	Raw264.7	Curcumin	SPIONs	Electroporation	RGERPPR peptide	The combination of the two therapies significantly improved the tumor inhibition effect	[73]
Macromolecule organic compound	4T1	Anti-miRNA-21, antimiRNA-10b	Polylactic acid-glycolic acid (PLGA)	Extrusion	uPA	Systemic administration showed a strong tumor bias and enhanced the combined antitumor effect of anti-mirNA-21 and anti-mirNA10b	[36]
	NK cells	let-7a	Polyamidoamine (PAMAM)	Incubation		Promoted tumor targeting and acted directly as antitumor agents without causing rejection	[74]
	Attenuated salmonella	Tegafur	Pluronic micelles F127	Extrusion	DSPE-PEG-RGD	Provided effective protective immunity and inhibited tumor metastasis to the lung	[75]
	SKOV3, PC3, HCT-116, Saos-2	Survivin-siRNA	Polyethylenimine (PEI) complex	Ultrasonic + incubation		The knockdown efficiency and storage stability of siRNA were improved, and tumor growth was significantly inhibited	[76]
	*E. coli* Trans T1	PBIBDF-BT + cisplatin	PEG–b-PLGA	Extrusion		Hitchhiked circulating neutrophils and accumulated at the tumor site, completely eradicating residual tumor after PTT	[69]
	Macrophage	DOX	PLGA	Extrusion	c-Met peptide	The cell uptake efficiency and antitumor efficacy of doxorubicin were significantly improved, resulting in enhanced inhibition of tumor growth	[77]
Metal nanoparticles	H22	DOX	Bi_2_Se_3_	Electroporation		Combining photothermal therapy with low-dose in vivo chemotherapy produced significant synergistic antitumor effects	[78]
	hpMSCs		Hollow gold nanoparticles (HGNs)	Incubation		Combined with the targeting ability of cell membranes and the infrared light absorption properties of HGN, HGN eliminated primary and metastatic tumors in vivo	[79]
	MCF-7		Ultrasmall 2D vanadium carbide quantum dots (V2C QDs)	Electroporation	Arg-Gly-Asp (RDG) peptide	With its biocompatibility, long cycle time, and endosomal escape ability, it can target cells and enter the nucleus to kill tumors	[80]
	DC2.4	DOX	HAuCl4	Incubation		Hyperthermia generated by near infrared light induced tumor ablation and triggered drug release, achieving chemophoration-photothermal combined therapy	[70]
	MDA-MB-231	Gelonin	Metal–Organic Frameworks (MOF)	Ultrasonic + extrusion		Protected proteins from protease digestion and escaped immune system clearance, and selectively targeted homotype tumor sites to promote uptake and voluntary release by tumor cells	[81]
Non-metallic nanoparticles	4T1	ICG + DOX	Mesoporous Silica Nanoparticles (MSNs)	Ultrasonic + extrusion		The synergistic effect of chemotherapy and photothermal therapy on breast cancer was realized	[82]
	MCF-7	DOX + Mefopanib hydrochloride	Pegylated MSN	Ultrasonic + extrusion		Improved the efficiency of tumor penetration and enhanced intracellular transport	[83]
	J774.A.1	ICG + catalase	Glutathione-responsive biodegradable silica nanoparticles	Ultrasonic + extrusion	AS1411 modification of cholesterol conjugate	Improved the hypoxic environment of tumors with blood–brain barrier penetration and cancer cell targeting ability	[84]
Protein	RAW 264.7	Paclitaxel	Albumin	Extrusion		Demonstrated prolonged blood circulation, selective accumulation, and improved antitumor efficacy	[85]
	4T1	Si-S100A4	Cationic bovine serum albumin (CBSA)	Incubation + extrusion		Protected siRNA from degradation, and showed good biocompatibility, significantly inhibiting the growth of malignant breast cancer cells	[68]
	Raw264.7		Laurate-functionalized Pt (IV) prodrug Pt (lau), human serum albumin (HSA) and lecithin	Ultrasonic		Treated with high-potency Pt chemotherapy for in situ tumors and pulmonary metastatic nodules	[67]
	RBC + MCF-7		Melanin granules	Ultrasonic + extrusion		The hybrid membrane nanoparticles had a long cycle, homologous tumor targeting ability, and inherited the photothermal properties and biocompatibility of the melanin core	[86]
Liposome	Mouse platelets	Glucose oxidase + ferric ammonium	Photothermal sensitive liposomes (DPPC:DPPG:Cypate)	Ultrasonic + extrusion		Photothermal therapy produced hydroxyl radicals that can be targeted with enhanced laser irradiation	[87]
	Sk-hep1	siCDK1	DPPC	Electroporation + extrusion		Mixed membrane vesicles derived from tumor cells enhanced tumor targeting	[88]
	DC2.4		CLT-loaded pegylated liposome	Ultrasonic + extrusion		KRAS mutated tumor cells showed enhanced pinocytosis	[89]
	CT26	R837	Photothermal sensitive liposomes (DPPC, DSPE-PEG_2000_, MSPC)	Ultrasonic + extrusion		Enhanced circulatory capacity combined with photothermal therapy and immune adjuvants	[27]
	4T1	BMS202 (BMS)	IR780-modified lipid NPs	Solvent diffusion method + extrusion		Stimulated the immune system in response to heat and light	[90]

**Fig. 4:**
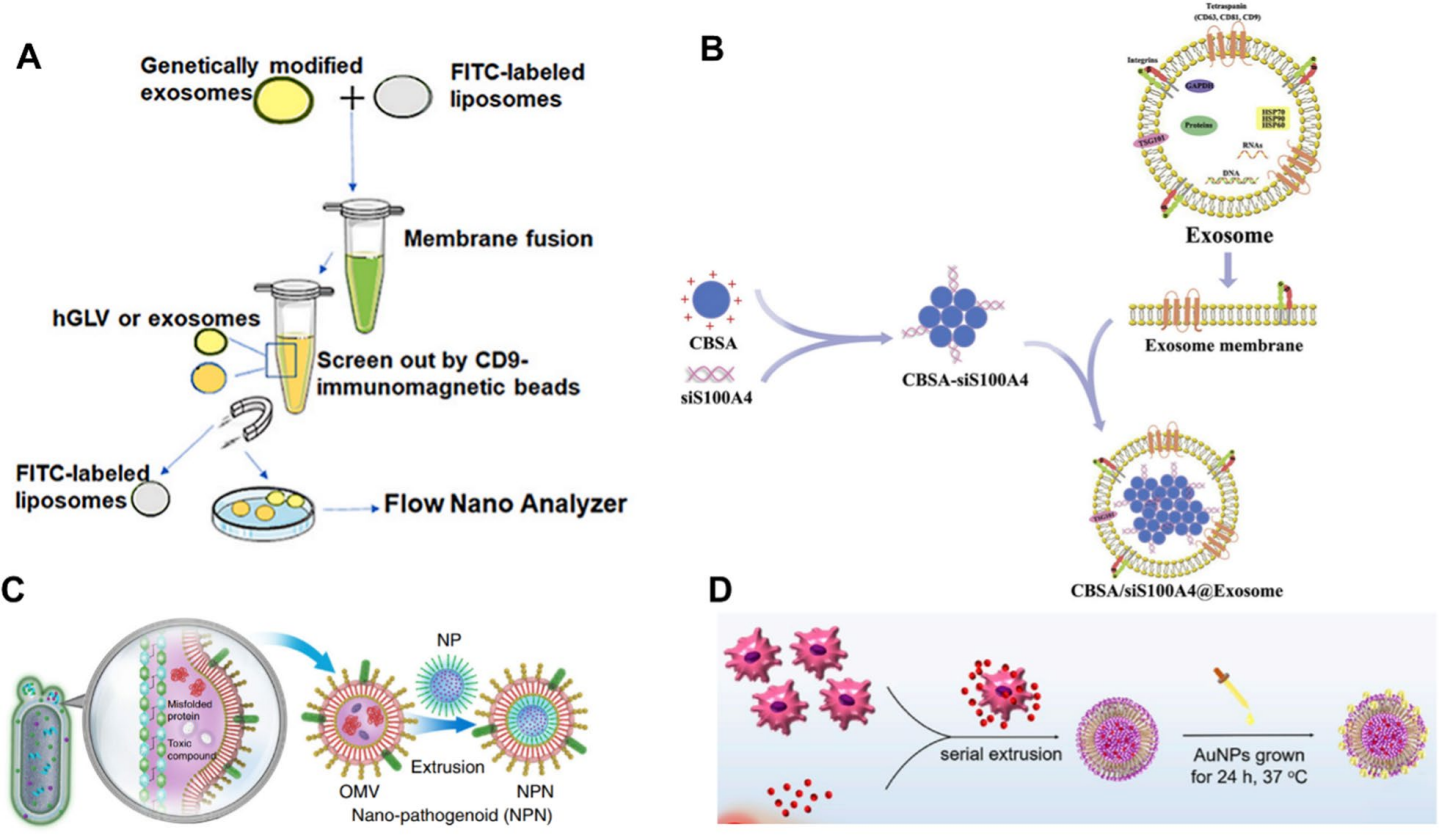
Example of fusion of membrane nanostructures with synthetic materials. **A** Schematic diagram of the method for preparing the gene-engineered exosome-liposome hybrid nanovesicles (hGLV) [27]. Reprinted with permission. Copyright 2021, Elsevier. **B** Cationic bovine serum albumin (CBSA)-coupled siS100A4 was coated with exosome membrane by extrusion to form biomimetic nanoparticles [68]. Reprinted with permission. Copyright 2020, Elsevier. **C** Nano-pathogenoids (NPNs) are prepared by coating Outer membrane vesicles (OMV) on nanoparticles NPs, and NPNs inherit pathogen-associated molecular patterns (PAMPs) from OMV [69]. Reprinted with permission. Copyright 2020, Nature Communications. **D** DC2.4 was mixed with DOX and extruded with polycarbonate films by a micro-extruder [70]. The obtained EVdox was incubated with HAuCl4·3H2O at 37 °C for 24 h to obtain EVdox@AuNP. Reprinted with permission. Copyright 2019, Elsevier

Inorganic materials such as gold, iron, and silica wrapped in the contents of cell membranes have unique physical, electrical, magnetic, and optical properties that can be used for diagnostic, imaging, and photothermal therapy (PTT) applications [61, 62]. Most inorganic nanoparticles have good biocompatibility and stability. However, their clinical use is restricted due to low solubility and toxicity issues [63]. The use of cell membrane as a shell facilitates long-term blood circulation and targeted delivery of nanoparticles in vivo and is not affected by the properties of the core nanomaterials [64]. Polymeric nanoparticles have favorable biodegradability, water solubility, biocompatibility, mimicability, and storage stability, which are also ideal materials for the targeted delivery of small molecules, biomacromolecules, proteins, and vaccines [65]. By adjusting the composition, stability, reactivity, and surface charge of the particles, the encapsulation and release of drugs can be precisely controlled. Their surfaces can also be easily modified with ligands to deliver drugs, proteins, and genetic material to target tissues. However, only a small number of polymeric nanomaterials have been approved by the US Food and Drug Administration (FDA) for clinical use, due to the agglomeration and non-specific clearance of nanoparticles. On the other hand, the insufficient drug carrying capacity of cell membrane vesicles is one of the reasons limiting their further application in drug delivery. The integration of polymeric nanoparticles with cell membrane not only retain the excellent biocompatibility and targeting ability of the original cell membrane, but also overcome the defect of insufficient drug loading. Lipid nanoparticles are the most common class of nanomedicines approved by the FDA, which have the advantages of simple formulation, self-assembly, biocompatibility, high bioavailability, and high drug loading [66]. Similarly, lipid nanoparticles can be fused with cell membranes to prepare corresponding membrane-coated EBPs. In addition, drug-carrying components from other biological sources can also be encapsulated as the contents of membrane-coated EBPs. For example, albumin is the protein with the highest content in plasma which has good biocompatibility, nonimmunogenicity, easy purification, and good water solubility. Although albumin nanoparticles can efficiently load hydrophobic drugs due to multiple drug binding sites, they may react with proteins in the blood, resulting in reduced uptake of nanoparticles by tumor cells. Xiong et al. showed that the Pt (IV) precursor was able to stably bind to albumin with the aid of lecithin, while the encapsulation of macrophage membranes further enhanced the ability of albumin nanoparticles to actively aggregate at the tumor site and effectively promoted the cellular internalization of drugs, where Pt(IV) precursor was decomposed into toxic Pt(II) drugs under cytoplasmic reduction [67]. This strategy promotes cellular internalization and the conversion of the precursor into its active form, improving the therapeutic potential of the drug. Figure 4 provides a concise overview of the methods employed for the fusion of membrane nanostructures with synthetic materials.

### Direct modification

**Fig. 5:**
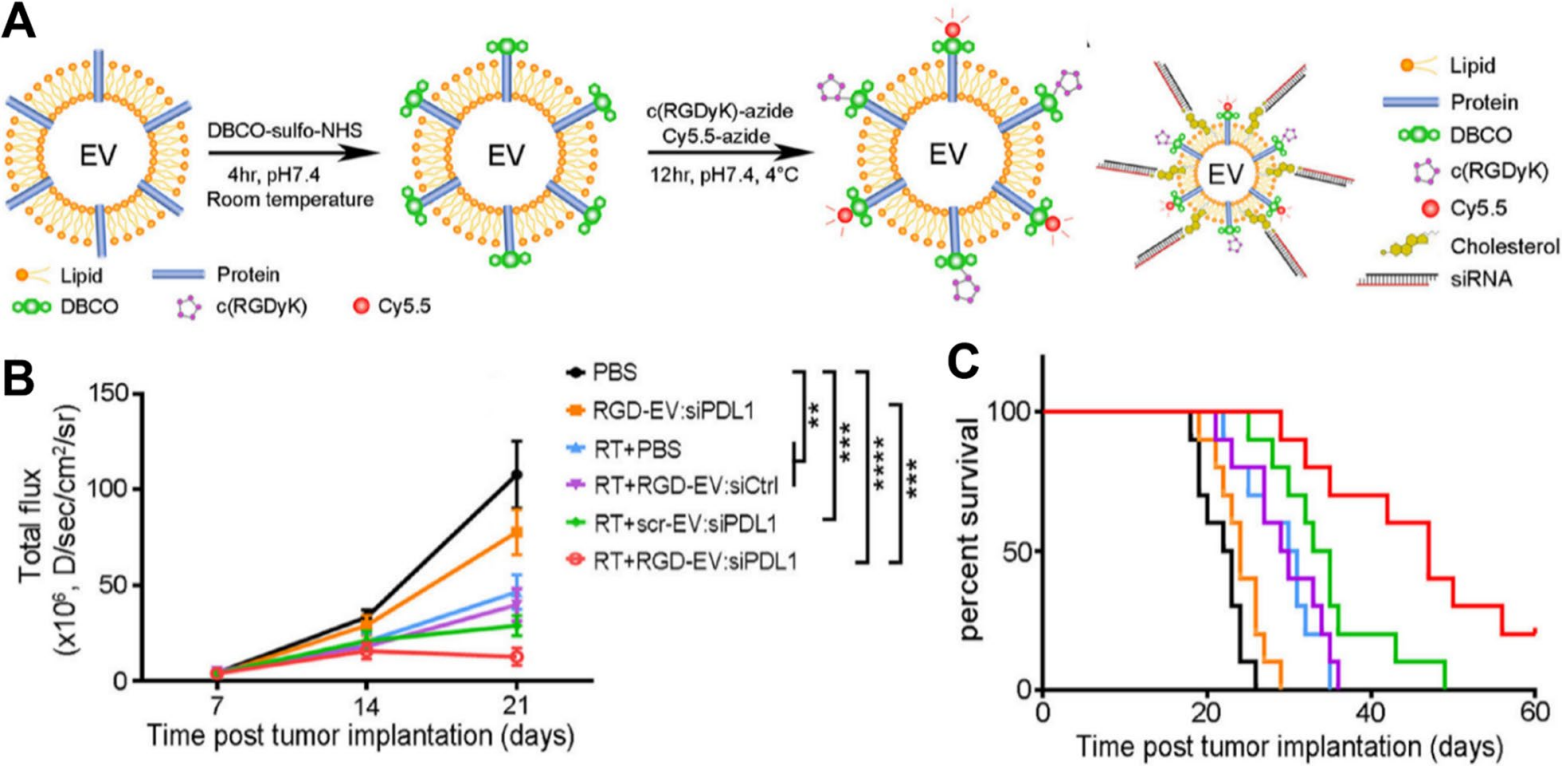
Schematic diagram of siRNA-loaded EVs conjugated with c(RGDyK) and their anticancer effects. **A** A diagram illustrating a two-step reaction that involves conjugating c(RGDyK) and Cy5.5 fluorophore or cholesterol-modified siPDL1 to the amine group of EVs. **B** Quantification of tumor-associated fluorescence radiance intensity with data presented as the mean ± SEM; **P < 0.01, ***P < 0.001, ****P < 0.0001, by one-way ANOVA. **C** Kaplan–Meier survival curves are shown (n = 10) [91]. Reprinted with permission. Copyright 2022, American Chemical Society

In addition, direct modification is also an artificial engineering strategy. There are more concerns about direct modification of EBPs than liposomes because they must be modified without compromising the integrity of vesicles. “Click chemistry” reactions can form chemical bonds under very mild conditions and are utilized to covalently bind large biomolecules. This method can also anchor the targeted portion to the cell membrane, controlling the structure and density of the targeted ligand regardless of the cell of origin. In addition, the reaction can be performed during the purification of vesicles, so it is more suitable for clinical applications than the genetic engineering approach. Tian et al. adopted a copper-free click chemistry method to conjugate cyclic (Arg-Gly-Asp-d-Tyr-Lys) peptide [c(RGDyK)] into cerebrovascular endothelial cell-derived EBPs (Fig. 5). EBPs-mediated siPDL1 targeted delivery successfully reversed the expression of radiation-stimulated PD-L1 in tumor cells [91].

Peptides as tumor cell targeting ligands have lower immunogenicity than antibodies, and researchers can use peptide libraries to screen for different tumor targets. For example, the small molecule polypeptide cNGQGEQc (CC8) that can bind to non-small cell lung cancer is a short peptide consisting of eight amino acid residues, which was first screened by Guo et al. using combinatorial chemical peptide library technology [92]. They added free thiols to both ends of cysteine residues in CC8 to help it modify the membrane through simple incubation [93]. This modification improved the short half-life and poor biological distribution of Imperaline, and played a targeting role in NSCLC cells. This work not only provides a targeted drug delivery system for Imperaline but also offers a new platform for targeting non-small cell lung cancer. Nied et al. produced another type of engineered EBPs for cancer treatment through direct modification. First of all, macrophages were polarized by Mn into anti-tumor M1 macrophages. Then, the azide group was introduced into cholinophospholipids on the cell membrane. The azide-modified M1 EVs were linked with the anti-CD47 antibody (aCD47) and anti-signal regulatory protein α (SIRPα) antibody through dibenzocycloctane (DBCO) and the pH-sensitive benz-imide bond [94]. This resulting M1 type EBPs were able to eliminate the “don’t eat me” signal and improve the phagocytosis ability of macrophages. In addition, the EBPs can effectively restore macrophages in the original tumor from M2 to M1, exerting a synergistic antitumor effect. This engineering strategy can be popularized and adapted to various environments, and the approach is relatively simple and easy to implement.

**Fig. 6:**
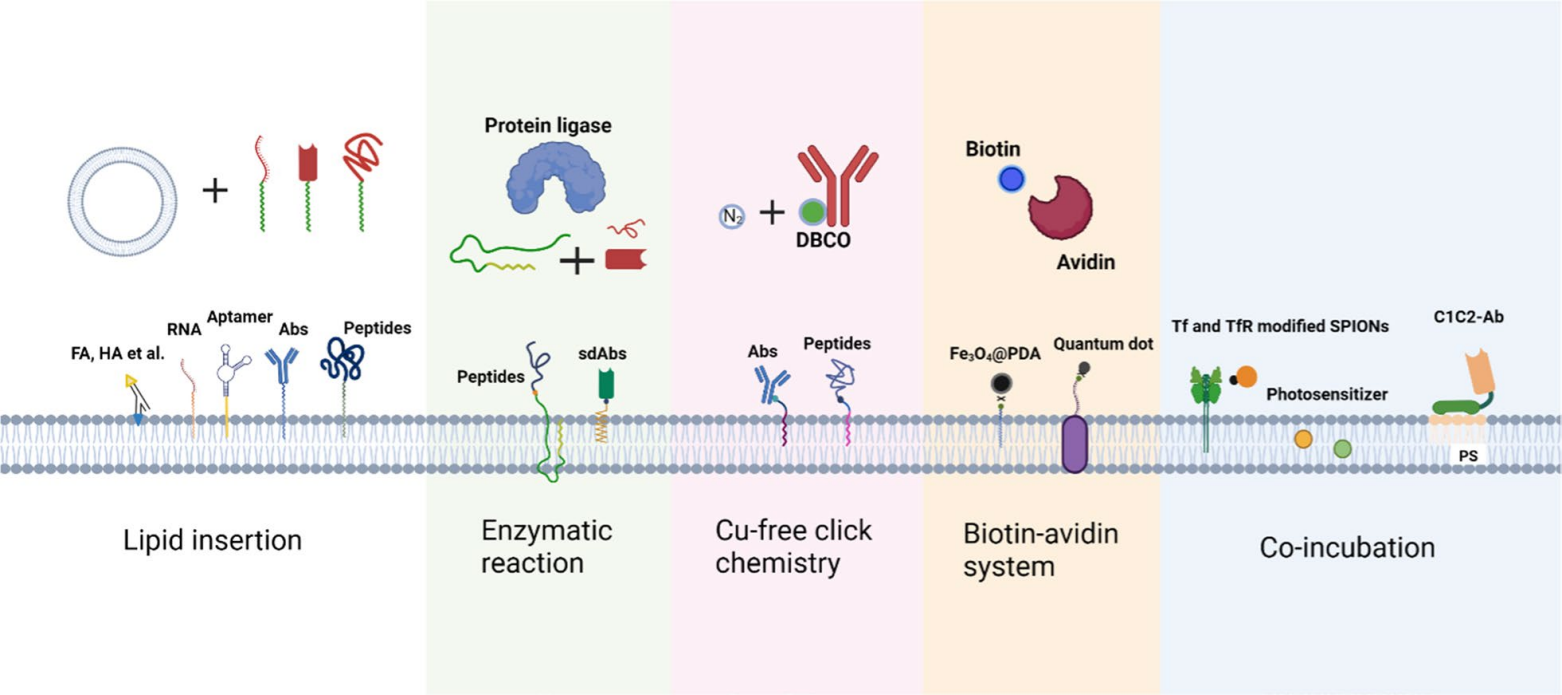
Schematic illustration of the direct modification of EBPs on the membrane surface. Examples of direct engineering modification of the surface of EBPs include integrating lipid-conjugated molecules into the lipid bilayer, utilizing click chemistry and affinity interactions to attach target molecules, relying on enzymatic covalent binding through peptides or antibodies, and using biotin-avidin reactions to bind specific molecules. *FA* folic acid, *HA* hualuronic acid, *Abs* antibodies, *DBCO* dibenzocyclooctyne, *PDA* polydopamine, *Tf* transferrin, *TfR* transferrin receptor, *SPIONs* superparamagnetic iron oxide nanoparticles

While modifying the surface of EBPs, precautions must be taken to avoid membrane rupture, surface protein denaturation, and changes in osmotic pressure resulting from exposure to excessive temperature, pressure, solvents, or low or high salt concentrations. Moreover, chemical manipulation may lead to surface protein inactivation or nanoparticle aggregation. Apart from covalent modifications, there are promising noncovalent methods for stable biofilm modification, such as membrane insertion using lipid-binding ligands or hydrophobic drugs, receptor-ligand binding, and transient membrane permeability via electroporation. Figure 6 illustrates the various functional structures that may be directly modified onto the membrane’s surface, including large molecular proteins, antibodies, and small molecular inorganic compounds. In addition, the modification types of EBPs are further diversified through multiple chemical reaction pathways.

It is a simple and direct method to modify functional molecules with lipophilic molecules and insert them into the cell membrane. DSPE-PEG, as a PEG phospholipid, has good biocompatibility and biodegradability and is often used as a nanoparticle carrier material to assist drug release, which can improve the biocompatibility and stability of the preparations [95, 96]. Functionalized DSPE-PEG can be used to modify proteins, peptides and other materials or small molecules containing sulfhydryl groups and then inserted into the bilayer phospholipid membrane through the DSPE terminal to obtain a new class of targeted EBPs. Lipophilic chemical chains and biofunctional nucleic acid compounds can also be modified onto the surface of EBPs by simple incubation methods. For example, valent-controlled tetrahedral DNA nanostructures (TDNs) were conjugated with DNA aptamers and loaded onto the surface of EBPs via cholesterol anchoring to achieve specific cell targeting [97]. EBPs carrying TLS11a aptamers that bind specifically to HepG2 cells and CRISPR-Cas9 RNA-directed endonucleases (RNPs) led to the downregulation of GFP or WNT10B in specific cells. Cholesterol binds to RNA in a similar manner to DNA. In the study conducted by Zhan et al. cPLA2 siRNA, an inhibitor of energy metabolism, binds to the cell membrane through the cholesterol chain, enabling a straightforward process to form a dual inhibitor with metformin [98]. Due to the active uptake of vesicles by GBM cells, drugs can be selectively concentrated at the tumor site without the need for external targeting design.

In addition, proteins or peptides can also be bound to the membrane surface of EBPs by protein ligases. In the study of Peng et al. the vesicles produced by erythrocytes were linked to the biotin-TRNGL enzyme, and biotin-TRNGL was sequentially coupled to a biotinylated linking peptide with streptavidin tetracer and biotinylated anti-EGFR nanoparticles (α-EGFR-VHH) to functionalize the surface of EBPs, which activated the innate immune system through the RIG-I receptor (RLR) pathway for anticancer treatment [99]. Pham et al. utilized Sortase A and OaAEP1 ligases to establish a permanent covalent bond between vesicles and peptides, and this catalytic reaction occurred at neutral pH. The resulting targeted EBPs significantly inhibited tumor growth by delivering low-dose paclitaxel (10–20 times lower than the clinical equivalent dose) [100]. This simple enzymatic method enables the coupling of peptides and nanoantibodies to the membrane without any genetic or chemical modification of the donor cell.

Gram-negative bacteria-derived outer-membrane vesicles (OMVs) hold significant potential as anticancer therapeutic agents in nanotechnology applications. However, OMVs’ intravenous injection can result in high toxicity and antibody-dependent clearance. To address these challenges, Qing et al. coated the OMVs’ surface with a pH-sensitive calcium phosphate (CaP) shell, which neutralizes the acidic tumor microenvironment [28]. This neutralization provides several advantages, such as improving targeted drug delivery to tumor cells and reducing toxicity to healthy cells. Table 4 provides more details on the direct modification methods of EBPs.

**Table 4:** Direct modification of EBPs

Type	Sources	Cargoes	Loading methods	Modification	Methods	Therapeutic applications		Refs.
Folic acid	RAW264.7	Photosensitizer protoporphyrin X (PpIX) + DOX	Incubation	Folic acid (FA)	Incubate with DSPE-PEG-FA	Focusing on tumor tissue	Prodrug conversion combined with photodynamic therapy	[101]
	E.coli BL21	ICG	Incubation	Calcium phosphate nanoshell (FA modified)	Incubate with calcium chloride solution		The PH-sensitive shell responded to the tumor environment	[28]
	HEK293T			FA-3WJ-survivin siRNA-A647	Incubate with DSPE-PEG-FA		siRNA carried by vesicles can avoid being captured by inclusions	[102]
	HT29	DOX	Incubation	EpCAM-MNPs/DSPE-PEG-FA	Incubate with DSPE-PEG-FA		Improved biocompatibility, induced hyperthermia and inhibited tumor growth	[103]
	Colostrum	Paclitaxel	Incubation	FA	Activated FA by EDC and NHS covalently attaching		Overall efficacy and safety were improved compared with traditional administration	[17]
	HEK293T	ICG	Incubation	FA	Activated FA by EDC and NHS covalently attaching		Sonodynamic therapy for antitumor	[104]
Polypeptide	ReNcell VM	Cholesterol-conjugated siPDL1	Incubation	c(RGDyK) peptide	Copper free click chemistry	Targeted integrin αvβ3 on cerebrovascular endothelial cells		[91]
	Human plasma	Imperialine	Soluplus solubilization + ultrasonic	CC8	Incubate with CC8-Mal-PEG-CLT	Targeted integrin α3β1 overexpressed in non-small cell lung cancer		[93]
	Mouse blood			Multifunctional chimeric peptide	Ice-water bath	Cytoplasmic membrane targeting combined with nuclear targeting photodynamic therapy		[105]
	Nude mouse macrophages			Legumain-specific propeptide of melittin (legM) + cytotoxic soravtansine (DM4) prodrug	Incubate with DMPE-PEG	Targeted the tumor microenvironment and performed prodrug transformation		[106]
	L929	MTX		lipid-KLA-LDL peptide	Incubation	Promoted apoptosis and selectively bound BBB and GBM cell lines		[107]
	cisplatin-resistant human ovarian cancer cell line SKOV3-CDDP	Triptolide + miR497	Mix in CaCl_2_ solution	cRGD-modified liposome	Ultrasonic + extrusion	Overcame cisplatin resistance, promoted intracellular ROS production, and induced macrophage polarization from M2 to M1		[108]
	Raw264.7	SPION + curcumin	Electroporation	RGERPPR peptide (RGE)	Cycloaddition reaction of sulfonyl azide	Targeted glioma cells and tumor vascular endothelial cells		[73]
Antibody	Raw264.7	AB680	Extrusion	aPDL1-PEG-DSPE	Incubation	Anti-PD-L1 therapy combined with CD73 inhibitors had a stronger anticancer effect		[109]
	HEK293T	HChrR6 mRNA	Transfection	Extracellular vesicle HER2-binding (EVHB) protein	Incubation	Showed high tumor targeting and protected the function of the prodrug activated mRNA		[110]
	Human erythrocyte	RIG-I agonist	Transfection	Anti-human EGFR antibody	OaAEP1 ligase + biotin/avidin reaction	Targeted tumors to activate the innate immune system through the RIG-I receptor (RLR) pathway for anticancer therapy		[99]
	M1 macrophage			CD47 and SIRPα antibodies	Azide-modified EBPs bind to dibenzocycloctyne modified antibodies of CD47 and SIRPα via ph-sensitive junctions	Blocked the inhibitory receptor SIRP-α on macrophages and CD47 on tumor cells		[94]
	HEK 293F	Microtubule polymerization inhibitor mertansine (DM1) + Verrucarin A (Ver-A)	Incubation + electroporation	anti-SSTR2 and anti-CXCR4 antibodies	DSPE-PEG-NHS linker	Targeted neuroendocrine tumors with chemotherapy drugs		[111]
	DC2.4			DSPE-PEG-NHS-aCD3/DSPE-PEG-NHS-aEGFR	Incubation	Promoted the proliferation and activation of T cells and mediated the cross-linking between T cells and B16-OVA cancer cells		[112]
	RBC/THP-1	Luciferase mRNA/paclitaxel	REG1 loading reagent + Ultrasonic	EGFR homing peptide	Protein ligases	Low dose targeted delivery of chemotherapy drugs		[100]
	RAW264.7 + mouse bone marrow macrophages	Paclitaxel	Ultrasonic	Aminoethylanisamide-polyethylene glycol (AA-PEG)	Ultrasonic with DSPE-PEG-AA	Targeted tumor tissue to improve circulation and the capacity of nanoparticles		
	HEK293T	Verrucarin A	Incubation	anti-EGFR antibody	DSPE-PEG-NHS linker	Had the ability to cross the blood–brain barrier, the specificity of tumor targeting, and the advantages of providing chemotherapy, gene therapy and other combined therapies		[113]
Photosensitizers	MIA-PaCa-2			Ce6	Ultrasonic	Photodynamic therapy and photoacoustic imaging function		[114]
	M1 macrophage	Hydrophilic hypoxia-activated prodrug AQ4N	Incubation	Carbopentoxyphenyl oxalate (CPPO) + chlorin e6 (Ce6)	Incubation	Chemical secondary sources produced active oxygen activated the photosensitizers for photodynamic therapy to promote prodrug conversion		[115]
	M1 macrophage	Doxubicin prodrug	Electroporation	Carbopentoxyphenyl oxalate (CPPO) + chlorin e6 (Ce6)	Incubation	Chemical energy generated by CPPO and increased H2O2 can directly activate photosensitizer Ce6 without photoexcitation		[116]
	B16F10			Zinc phthalocyanine	Incubation	Photodynamic therapy, combined with cell membrane, can increase the stability of the photosensitizer solubility		[117]
Nucleic acid	J774.A.1	Glutathione-responsive biodegradable silica nanoparticles	Ultrasonic + extrusion	Cholesterol conjugated AS1411	Incubation	Penetrated BBB and targeted cancer cells, alleviated hypoxia within tumors, and improved the efficiency of sonodynamic therapy		[84]
	HEK293T	Cas9 RNP	Freeze thawing / Ultrasonic	Tetrahedral DNA nanostructures (TDN) + TLS11a aptamer	Incubation	Tumor specific targeting and delivery of CRISPR/Cas9		[97]
	Serum	DOX	Incubation	SPIONs-Tf + chol-miR21i + endosomolytic peptides L17E	Incubation	Improved the escape of endosomes and effectively delivered cargo to tumor cells		[118]
	HeLa	AS1411-TMPyP4 + ICG	Incubation	Chol-Sgc8	Incubation	Targeted high expression of protein tyrosine kinase 7 (PTK7) in tumor cells		[119]
	Mouse DC cell	Paclitaxel	Ultrasonic	AS1411	Incubate with AS1411-PEG 2000—Chol	Rapid and simple preparation of tumor targeting drugs		[20]
Metal particles	MSCs			SPIONs/ Cell penetrating peptide (CPP) and TNF-α	Incubation/ Transfection	Enhanced tumor targeting and inhibited tumor growth under an external magnetic field		[51]
	THP-1	DOX	Electroporation	Polydopamine (PDA) coated magnetic Fe3O4 nanoparticles (Fe3O4@PDA)	Incubation by Biotin-avidin method	Magnetic field guided tumor targeting using chemical, genetic, and photothermal methods resulted in a significant reduction in tumor size		[120]
	Neutrophils	DOX	Extrusion with DOX loaded cationic liposome	Superparamagnetic iron oxide nanoparticles (SPIONs)	Incubation	Dual tumor targeting capabilities and loading chemotherapy agents		[121]
Chemotherapeutic drugs	BM-MSCs	Galectin-9 siRNA	Electroporation	Oxaliplatin modified with maleimide (OXA-MAL)	Vortex	Induction of tumor inhibition by macrophage polarization, recruitment of cytotoxic T lymphocytes, and down-regulation of Tregs induced antitumor immunity		[122]
Mannose	DC2.4 + human serum	DOX	Ammonium sulfate gradient method	c(RGDm7)-LS-GE + cationic mannan	Extrusion/ incubation	Saturated macrophage receptors and reduced liver accumulation for subsequent administration		[123]
	MSCs	AntagomiR-182 inhibitor	Electroporation	Mannan	Incubation	Targeted macrophages and reversed the immunosuppressive environment		[124]
	*E. coli* BL21 (ΔmsbB)	Redd1 siRNA	Electroporation	DSPE-PEG-CA-PTX和DSPE-PEG-mannose	Incubation	Targeted macrophages and regulated the tumor microenvironment		[125]
Hyaluronic acid	RAW 264.7	DOX	Ultrasonic	Hyaluronic acid	Ultrasonic	Targeting CD44, which is highly expressed on the surface of tumor cells		[126]
Quantum dot	M1 Raw264.7	DOX + hairpin DNA probe	Electroporation	Quantum dot labelling	DNA chain connection	A combination of biotherapeutic and chemotherapy functions and bioimaging capabilities		[127]
DSPE-PEG	*Asparagus cochinchinensis*			DSPE-PEG	Vortex + ultrasonic	Pegylated nanoparticles can better inhibit tumor growth without significant side effects		[16]

## Application of engineered EBPs in cancer therapy

### Enhancing tumor targeting capabilities

**Fig. 7:**
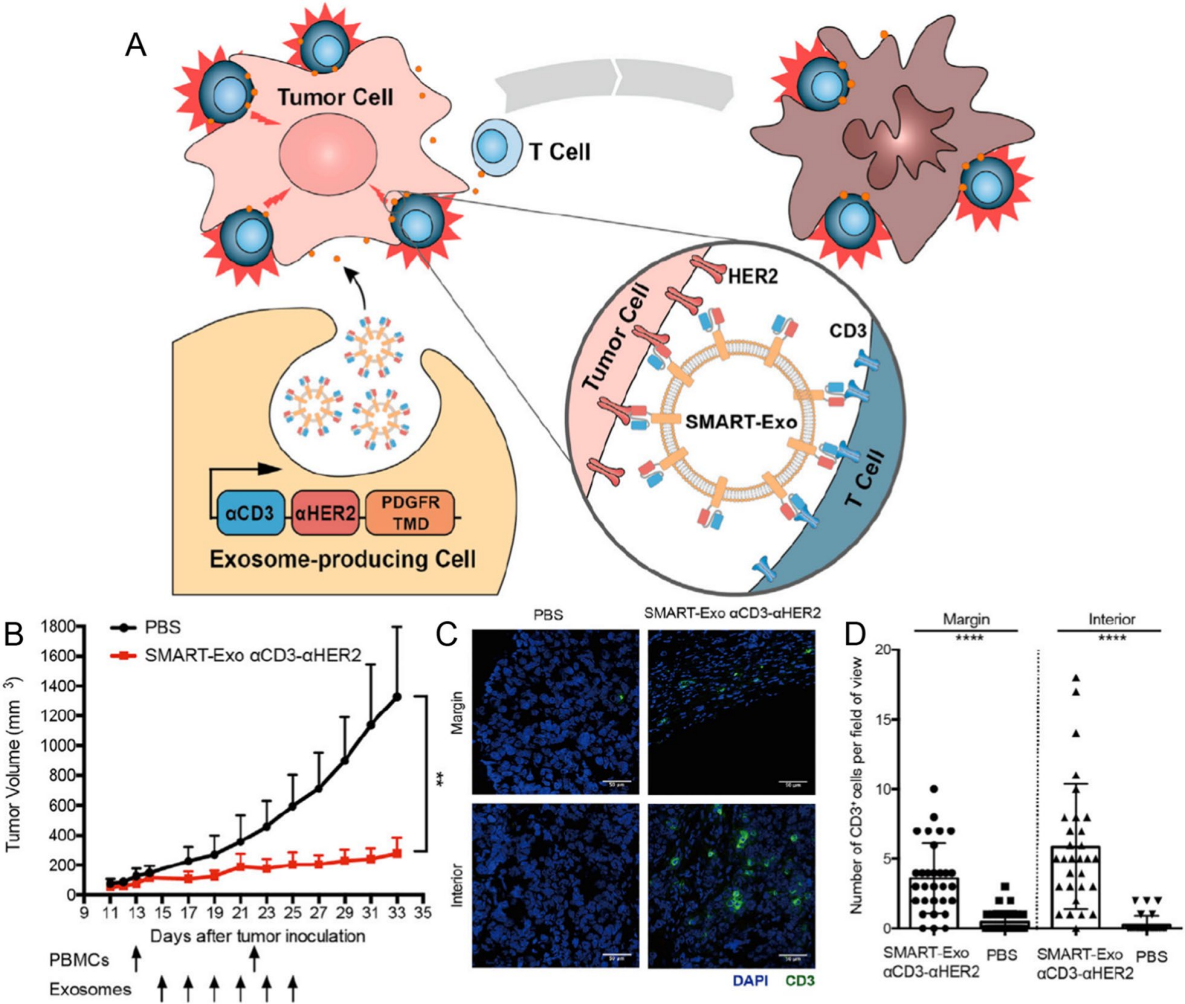
Schematic of SMART-Exos and their anticancer efficacy. **A** The design and application of αCD3-αHER2 SMART-Exos as a targeted immunotherapy for breast cancer. **B** The in vivo antitumor effect of αCD3-αHER2 SMART-Exos. **C** Representative immunohistofluorescence images of the margin and interior of frozen tumor sections from PBS- and SMART-Exos-treated mice. Blue: nuclei stained with DAPI. Green: CD3 + cells stained with the anti-CD3 antibody. Scale bars: 50 μm. **D** Quantitative representation of the number of CD3 + cells from each field of view along the margin and interior of each tumor from PBS- and SMART-Exos-treated groups (15 fields of view per region and two mice per group) [38]. Reproduced with permission. Copyright 2020, Elsevier. *PDGFR* platelet-derived growth factor receptor, *TMD* transmembrane domain, *SMART-Exos* synthetic multivalent antibodies retargeted exosome, *HER2* human epidermal growth factor receptor 2

One common strategy to enhance the tumor targeting of nanoparticles is through modification with tumor-specific ligands. Human epidermal growth factor receptor-2 (HER2) is among the most widely examined breast cancer genes and is a prognostic indicator for clinical treatment monitoring that plays a crucial role in targeted therapy. Shi et al. successfully generated dual-targeted EBPs that targeted T-cell CD3 and tumor HER2 receptors via simultaneous overexpression of anti-human CD3 and anti-human HER2 antibodies (Fig. 7). This technology brought human T cells to HER2-positive breast cancer cells, thereby inducing a highly focused and tumor-specific immune response. [38]. Furthermore, the potential of targeted EBPs was further enhanced by their ability to carry RNA or chemotherapeutic drugs, thus maximizing the therapeutic impact. Targeted EBPs can also be loaded with RNA or chemotherapeutic drugs to further improve the therapeutic effect. EBPs targeting the HER2 receptor and loaded with HChrR6 mRNA in combination with *6-chloro-9-nitro-5-oxo-5H-benzo[a]phenoxazine* (CNOB) can specifically kill HER2-positive cells [40].

Such therapeutic strategies are increasingly being applied to other common tumor targets. The urokinase plasminogen activator receptor (uPAR) is the main receptor of the plasminogen activation system, which is involved in the activation of plasminogen. EBPs overexpressing urokinase-type plasminogen activating peptide (uPA pep) can target uPAR and participate in specific tumor targeting [36]. The combination of uPAR targeting and individual anti-miRNA-loaded nanoagents mediates significant improvements in anticancer therapy’s tumor therapeutic efficacy. The targeted synergy of multiple cell signaling pathways results in a lower therapeutic dose required for each anti-miRNA or chemotherapy agent. Glypican3 (GPC3), a heparan sulfate glycoprotein located on the surface of cell membranes, is a specific antigen associated with cancer. Anti-GPC3 ScFV-modified EBPs deliver miR-26a to GPC3-expressing hepatocellular carcinoma (HCC) cells [37]. These approaches capitalize on the specific characteristics of cancer cells, such as the overexpression of uPAR and the presence of cancer-associated antigens like GPC3, to enable precise and efficient tumor targeting, opening up new avenues for more effective and personalized cancer treatments.

In addition to large molecules such as proteins or antibodies, some small molecules can be used to decorate the surface of the EBPs in a more flexible way. Folic acid receptor (FR) is widely expressed on the surface of a variety of tumor cells. Compared with normal lung tissue, FR is highly expressed in tumor tissue (100 times) and only slightly expressed in normal cells. In addition to the method of binding the DSPE-PEG chain and then inserting it into the cell membrane, folic acid (FA) can also be bound to the cell surface by chemical reaction-mediated covalent binding. The mitochondrial heme synthesis pathway can efficiently convert 5-aminolevulinic acid (5-ALA) into the photosensitizer PpIX. By adopting this principle, Li et al. allowed cells to take up 5-ALA and then converted it into photosensitized agents for packaging into vesicles, which could not only overcome the low drug loading efficiency of the passive encapsulation method but also avoid the destruction of membrane integrity caused by the active encapsulation method [101]. FA-modified macrophage-derived vesicles further realized the targeted delivery of anticancer drugs. Although cancer cell-derived EVs show high tumor targeting and internalization effects, considering the safety of clinical applications, the use of normal cell lines and simulated tumor cell membrane components may be an effective way to improve tumor targeting. Based on the observation of a higher content of phosphatidylcholine (PC) in the EVs of glioma cells, PC molecules were inserted into the membrane lipid layer of reticulocyte-derived EVs using a simple incubation method. It was found that the resulting EBPs significantly promoted the accumulation of loaded drugs in tumor cells [128].

Large proteins such as antibodies can be displayed on the surface of vesicles without genetic engineering. As mentioned earlier, DSPE-PEG chains can not only modify small molecular compounds such as folic acid but also serve as links for proteins and peptides to be inserted into cell membranes. The terminal groups of PEG can be activated and linked to various targeting ligands, such as anti-PD-L1 antibodies, which are subsequently inserted into the outer membrane of EBPs through the lipid accumulation of DSPE [109]. Since anti-PD-1/PD-L1 alone may increase the expression of CD73 on cancer cells, which will induce immunosuppression and reduce the therapeutic effect, the combination of PD-1 antibody and CD73 inhibitor (AB680) co-delivered by EBPs has achieved a synergistic antitumor effect. In another study, Si et al. linked DSPE-PEG-NHS with anti-SSTR2 monoclonal antibodies and anti-CXCR4 monoclonal antibodies to target neuroendocrine tumors, and mPEG-DSPE acted as a stabilizer to improve the circulation stability of EBPs [111].

In addition to tumor cells, immune cells surrounding tumors are also popular targets for drug delivery. For example, mannose and mannose receptor (MR) pairs can recognize and capture antigen molecules, promote antigen presentation, and regulate the maturation and differentiation of immune cells. Given that both tumor cells and macrophages overexpress MR on their surfaces, mannose modification or mannosylation is an effective dual targeting strategy that can be involved in the design of specific nanomaterials. OMV comes from microorganisms, which are more easily recognized and phagocytosed by macrophages. In a study conducted by Guo et al. they demonstrated that OMVs modified with mannose exhibited excellent targeting ability towards a specific subtype of macrophages called M2-type macrophages. These M2-type macrophages are known to be present in the tumor microenvironment and have been implicated in promoting tumor progression [125]. When OMVs were modified with mannose, they specifically targeted and interacted with M2-type macrophages. This interaction led to the downregulation of Redd1. Additionally, the mannose-modified OMVs increased the levels of glycolysis, a metabolic process that provides energy to cells. This alteration in the metabolic phenotype of macrophages was crucial in transitioning their function from promoting tumor progression to inhibiting tumor progression. By modifying OMVs with specific molecules such as mannose, they can be engineered to selectively target specific cell types, such as M2-type macrophages. This targeted delivery approach holds great promise for harnessing the immune system to combat tumor growth and offers new avenues for developing innovative and effective cancer treatments.

Magnetic nanoparticles, together with an external magnetic field and/or magnetized implants, can deliver and anchor the particles to the target area, enabling local and sustained drug release. Hyperthermia refers to placing superparamagnetic ferric oxide in an AC electromagnetic field, which can make the magnetic direction randomly change between parallel and antiparallel so that magnetic energy can be transferred to particles in the form of heat, a property that can be utilized in the body to destroy sick cells. Iron oxide particles not only have magnetic responsiveness but also regulate the tumor microenvironment and trigger T-cell recruitment when combined with thermochemotherapy. Bose et al. successfully wrapped gold-iron oxide nanoparticles (GION) into EBPs derived from tumor cells to deliver therapeutic anti-Mir-21 and doxorubicin (DOX) drugs to cancer cells, achieving superior anticancer effects [71]. DOX was loaded into EBPs by fusing the positively charged liposomes carrying DOX with cell membrane vesicles. In addition, these EBPs can also be used as contrast agents for magnetic resonance imaging (MRI) and computed tomography (CT). Shen et al. also recently demonstrated that iron oxide nanoparticles wrapped in cell membranes show promising tumor targeting and stimulate T-cell infiltration with the help of magnetic fields, effectively inhibiting tumor growth [72].

### Improving anticancer efficacy

In addition to enhancing targeting capabilities, the engineering strategies may also improve the anticancer effects of EBPs. Tumor necrosis factor-associated apoptosis inducing ligand (TRAIL) is a membrane protein that can induce tumor cell apoptosis with tumor targeting. Shi et al. developed EBPs using amphiphilic poly(lactic-co-glycolic acid) (PLGA) carrying oxaliplatin (OXA) and hydroxychloroquine as the core, and the cell outer membrane with TRAIL as the shell [49]. These EBPs demonstrated good antitumor therapeutic effects for both in situ and metastatic hepatocellular carcinoma. To address the toxicity issues associated with the systemic administration of TNF-α, Zhuang et al. fused the cell penetrating peptide with TNF-α to form a fusion protein that anchored TNF-α in the cell membrane. This approach produced TNF-α presenting vesicles that can kill cancer cells under the magnetic targeting of superparamagnetic iron oxide nanoparticles [50]. These studies collectively demonstrate the potential of EBPs for targeted cancer therapy [51].

**Fig. 8:**
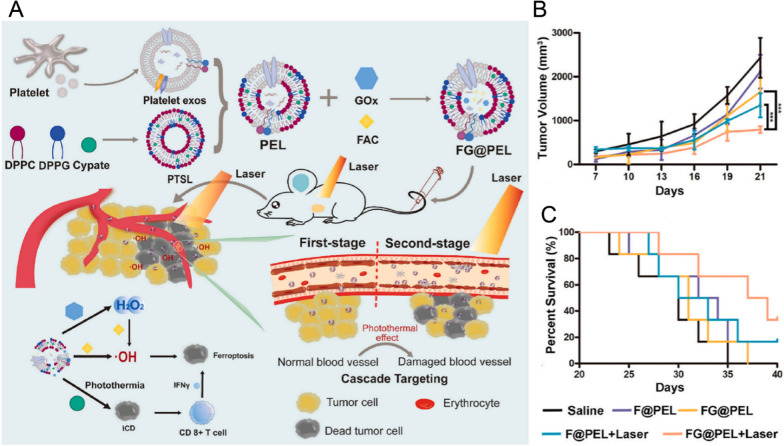
The combined application of two cascade treatment strategies based on engineered EBPs. **A** Schematic illustration of the structural design and therapeutic mechanism of photothermal EBPs combined with CDT. **B** The size of H22 tumors in mice following various treatments is depicted in terms of tumor volume. **C** The survival curve of H22 tumor-bearing mice after receiving different treatments [87]. Reproduced with permission. Copyright 2022, Elsevier. *PTSL* photothermal sensitive liposomes, *PEL* platelet exosomes with photothermal sensitive liposomes, *FAC* ferric ammonium. *Gox* glucose oxidase

The antitumor properties of EBPs can be activated or promoted by the application of external light or magnetic fields. For example, thermosensitive and photosensitive EBPs are promising nanocarriers that effectively utilize the dual advantages of EBPs and thermotherapy/photodynamic therapy (PDT) to improve therapeutic efficacy and reduce side effects. In addition, the increase in temperature can be combined with chemodynamic therapy (CDT), which is an emerging therapeutic approach that utilizes Fenton-type reactions to produce highly cytotoxic hydroxyl radicals that can resist the anoxic environment of tumors. The hydrogen peroxide generated by the reaction of glucose oxidase (GOx) with glucose can enhance the Fenton reaction at the tumor site. Photothermally sensitive EBPs can be used to produce photothermal effects under irradiation, and the increase in temperature can further enhance the activity of GOx (Fig. 8), while laser irradiation can also enhance the targeting ability of EBPs [87].

EBPs carrying a photosensitizer also act as photosensitive nanocarriers. Ce6 is usually highly efficient at producing singlet oxygen because it is suitable for the development of PDT for tumors. However, like most photosensitizers to date, Ce6 is hydrophobic and tends to aggregate in solution, so its practical application has encountered some difficulties. Hydrophobic Ce6 loaded into the lipid bilayer can provide photoacoustic imaging and PDT functions. CPPO is another type of chemical cold light source with high brightness and high efficiency of chemiluminescence. Wang et al. used these two hydrophobic agents (chemical excitation source CPPO and photosensitizing agent Ce6) to functionalize EBPs derived from M1 macrophages [115]. The intrinsic properties of M1-type macrophages allow EBPs to penetrate the blood–brain barrier and regulate the immunosuppressive tumor microenvironment through the conversion of M2-to-M1 polarization. The hydrogen peroxide produced in the process can be utilized to activate Ce6 and generate a large number of reactive oxygen species to achieve CDT. The exacerbation of tumor hypoxia also leads to the conversion of the nontoxic hydrophilic anoxic-activating prodrug AQ4N into toxic AQ4 for chemotherapy. Therefore, the EBPs achieved effective synergy between immunomodulation, CDT, and hypoxia-activated chemotherapy, exerting a powerful therapeutic effect on GBM.

Similar to photosensitizers, gold nanoparticles are also photoresponsive. The MSC membrane encapsulated hollow gold nanoparticles with a near-infrared (NIR) response, which showed enhanced nanoparticle delivery efficiency and tumor killing ability compared to conventional metal nanoparticles. Compared with traditional metal semiconductor quantum dots and organic dyes, carbon quantum dots not only maintain excellent fluorescence properties but also overcome the shortcomings of poor optical stability, high toxicity, and complex preparation processes. Cao et al. produced ultrasmall two-dimensional vanadium carbide quantum dots to show a strong photothermal effect at NIR light, and the EBPs prepared by wrapping them in tumor cell membranes exhibited good fluorescence imaging ability and photothermal effects in cancer treatment. In addition to being wrapped in the interior of EBPs, gold nanoparticles can also gather on the surface of EBPs to achieve photothermal conversion and kill tumors [80]. The use of VCQDs and AuNPs within the EBPs offers a multifunctional approach, combining fluorescence imaging, photothermal effects, and tumor targeting capabilities.

### Reshaping the immunosuppressive tumor microenvironment

Activating the antitumor immune response at the tumor site and improving the immune tolerance microenvironment are also hot research directions in cancer treatment. EBPs derived from tumor cells overexpressing the high-affinity variant human PD-1 protein (havPD-1) can block the function of highly expressed PD-L1 in tumors, induce tumor cell apoptosis, and activate cytotoxic T cells in combination with low-dose PARP inhibitors, thereby effectively treating homotypic tumors [52]. Liu et al. found that adenovirus infection promoted the presentation of MHC-I binding antigens on DCs and simultaneously increased the overexpression of anti-PD1 antibodies and B7 costimulatory molecules, so that EBPs derived from DC cells could activate the immune response and break immune tolerance [53]. In addition to delivering drugs, targeting tumor cells can also bring immune cells to the tumor site. EBPs derived from Expi293F cells expressing both CD3 and EGFR were able to bring CD3-expressing T cells to TNBC cells overexpressing EGFR [25]. The tumor antigen human papillomavirus type 16 early protein E7 (HPV16E7) is a major driving factor in the development and progression of cervical cancer. HPV16E7 was expressed in the membrane vesicles of Escherichia coli by genetic engineering technology for subcutaneous immunotherapy to induce an E7 antigen-specific cellular immune response [54].

In addition to directly activating immune cells, the high molecular weight hyaluronic acid (HA) around tumor tissues is also a barrier to the infiltration of immune cells. As a membrane protein, hyaluronase itself has the inherent ability to be expressed on the surface of cell membranes and can be simply modified to express on the surface of cell membranes. Considering that hyaluronidase can degrade high molecular weight HA to low molecular weight HA (LMW-HA), some studies have used this feature to polarize macrophages into the M1 phenotype by LMW-HA and reduce the number of associated immunosuppressive cells, thus changing the immune microenvironment from immunosuppressive to immunosupportive, reshaping the tumor microenvironment, and improving the efficacy of chemotherapy [57]. Prostaglandin F2 receptor negative regulatory factor (PTGFRN) can enhance drug delivery to tumor-associated macrophages (TAMs), and the PTGFRN-engineered EBPs effectively delivered STAT6-targeting ASO to TAMs and reprogrammed them into a proinflammatory M1 phenotype, which is beneficial for cancer treatment [58]. This approach shows promise in the field of cancer treatment by leveraging the immune response within the tumor microenvironment. By enhancing drug delivery to TAMs, it becomes possible to target and modulate their functions, with the goal of shifting them towards a proinflammatory M1 phenotype that is more beneficial for cancer treatment.

EBPs derived from bacteria have immunomodulatory activity, showing great potential for the development of anticancer drugs and nanotechnology applications. The outer membrane vesicles (OMVs) released by genetically engineered bacteria can serve as vaccine carriers to break autoimmune tolerance and induce the production of autoantibodies for cancer therapy. The BFGF antibody can effectively inhibit angiogenesis and tumor growth. The use of BFGF-modified OMVs (BFGF-OMVs) as an antitumor vaccine can induce high levels of anti-BFGF autoantibodies, which can inhibit angiogenesis, promote tumor cell apoptosis, and reverse tumor immunosuppression [55]. Virus-derived proteins also inherit some of the characteristics of the virus itself. Vesicular stomatitis virus glycoprotein (VSV-G), a fusion protein derived from the virus, can induce cell membrane fusion. The researchers expressed this protein on the surface of M1 macrophage-derived extracellular vesicles (M1 EVs) and then functionalized the EVs by loading siPD-L1. The resulting siRNA@V-M1 EVs can be targeted to tumors due to the natural targeting ability of M1 EVs. In the acidic tumor microenvironment, the pH-responsive VSV-G changed to an unfolded fusion state and then induced EVs to fuse with the targeted cell membrane, thus bypassing the endocytic uptake pathway [26].

PTT induces immunogenic cell death (ICD) through NIR-mediated photothermal ablation and release of tumor-associated antigen (TAA), which subsequently activates the immune response in vivo. Tan et al. selected dipalmitoyl phosphatidylcholine (DPPC) as a thermosensitive lipid component to construct thermosensitive lipid nanoparticles, incorporated the PD-1/PD-L1 inhibitor BMS202 into NIR-responsive EVs, and finally constructed thermosensitive EBPs by membrane fusion that can target both primary tumors and lung metastases [90]. Under laser irradiation, thermosensitive EBPs can influence the extracellular matrix in the tumor microenvironment and tumor-associated fibroblasts, thereby increasing the infiltration of tumor-infiltrating lymphocytes. Meanwhile, NIR radiation can trigger the reactive release of PD-1/PD-L1 inhibitors, block the PD-1/PD-L1 pathway, and reverse tumor immunosuppression. Exposed cationic lipid nanoparticles can capture tumor-associated antigens, thereby enhancing the maturation and antigen presentation of DCs and activating cytotoxic T lymphocytes to kill tumor cells. Therefore, the combined EBPs synergistically exerted an antitumor immune response to inhibit the growth and metastasis of primary tumors.

### Improving the utilization rate of drugs and reducing their toxic effects

Polymeric nanoparticles are commonly used drug as carriers in cancer treatment. The utilization of pH, thermal, ultrasonic, enzyme, and light-sensitive block copolymers allows for controlled micellar dissociation, triggering drug release. This approach not only enhances the specificity and effectiveness of micellar drug delivery but also provides insights into the development of smart drug delivery systems. By integrating EBPs with these industrial products, drug delivery systems with improved stability, higher drug-carrying capacity, and slower release kinetics can be achieved.

Chen et al. employed a novel polymerized non-ionic surfactant, Pluronic F-127, to encapsulate the drug Tegafol [75]. The stability of the micelles in the OMV coating was stronger, whereas the uncoated F127 micelles showed easy removal in an aqueous solution. The encapsulated F127 micelles exhibit spherical, dense nanostructures and can maintain a constant particle size in high serum and salt solutions. Encapsulated drugs may be rapidly released under acidic conditions, such as tumors or endosomal microenvironments. They further modified it with polyethylene glycol and RGD peptide to enhance its circulation and tumor targeting capabilities. This enabled its combination with bacterial immunotherapy and chemotherapy. PLGA [Poly(lactic-co-glycolic acid)] stands as the most representative type of nano-carrier system, consisting of lactide and glycolide copolymers [129]. It possesses excellent biocompatibility and biodegradability. At physiological pH levels, the hydrophobic core of PLGA contains an array of peptides and proteins, allowing encapsulated vesicles to bind and interact with the cell membrane. This ensures that peptides and proteins enter the cells through endocytosis without significant degradation. Li et al. demonstrated that OMV-encapsulated PEG-b-PLGA nanoparticles possess a typical core–shell structure and exhibit stability in a biological environment [69].

Dendrimers are capable of loading various types of drugs, including insoluble ones, in large quantities. Current research mainly focuses on nucleic acid and small molecule delivery. Both polymer types protect loaded therapeutic RNA, bind to the vesicle shell with targeted functionality, and exhibit effective tumor tissue killing effects. In the study by Wang et al. natural killer cell-derived exosomes (NKEXO) were utilized to encapsulate dendritic macromolecular cores for loading therapeutic miRNAs. Natural killer cells exhibit specific aggregation towards tumors without exerting cytotoxic effects on normal tissues. Simultaneously, the acidic tumor microenvironment facilitates the accumulation of these nanoparticles, thereby enhancing the biocompatibility and targeting capabilities of PAMAM dendrimers. Polyethylenimine (PEI), another widely used organic macromolecule, possesses a high cationic charge density and protonated amino nitrogen atoms. This facilitates the binding of negatively charged nucleic acid molecules, forming positively charged complex particles. Zhupanyn et al. developed an extracellular vesicle-modified PEI/siRNA complex. Upon intravenous administration in tumor-bearing mice, this complex showcased specific tumor inhibition through siRNA [76]. The modification of the PEI complex by extracellular vesicles altered key physicochemical and biological properties, enhancing nanoparticle functionality.

It is widely accepted that inorganic non-metallic nanoparticles are generally considered to have better biosafety compared to metal nanoparticles, along with higher drug loading capabilities. Among them, porous silicon nanoparticles exhibit excellent drug loading abilities, high biocompatibility, and biodegradability. In a study conducted by Wu et al. glutathione-responsive biodegradable silica nanoparticles were prepared to encapsulate catalase and ICG (Indocyanine Green). This fusion preparation not only enhances the efficacy of acousodynamic therapy but also maintains low toxicity to normal tissue [84]. Furthermore, engineered EBPs (extracellular vesicle-based particles) that wrap macrophage-derived EVs on their surface demonstrate effective penetration of the blood–brain barrier and accumulation at the tumor site.

Metal–organic frameworks (MOFs) are a type of organic–inorganic hybrid material composed of organic ligands and metal ions or clusters, self-assembled through coordination bonds to form intramolecular pores. MOF-protein (MP) nanoparticles possess a large internal surface area, high non-covalent affinity, and excellent drug loading capabilities. The pH-responsive metal–ligand bonds within MP nanoparticles, upon cellular internalization, can release proteins in acidic endosomes and lysosomes. This protects the proteins from protease digestion and immune system clearance and enables selective targeting of homologous tumor sites [81].

## Challenges in clinical translations

EBPs used as drug delivery systems in clinical trials need to fulfill the same basic requirements as traditional synthetic carriers, including simplicity, efficiency, and scalability [130]. During the large-scale production process, several factors need to be considered, such as the selection of production cell lines and media, whether to add serum or other treatments, routine or hypoxic environments, and two-dimensional or three-dimensional culture supports. It is preferable to use stable and safe cell sources with high secretion capacity, such as mesenchymal stem cells (MSCs) and HEK293 cells, to help reduce production costs and improve efficiency. Umbilical cord-derived multifunctional mesenchymal cells possess abundant clinical study data and high safety, making them the preferred choice for the production of EBPs. In addition, it is necessary to conduct a comprehensive composition analysis for all the EBPs used in clinical studies. Due to the potential inaccuracies in particle determination, we recommend the measurement of vesicle particle number or total protein mass as a dosing standard and the quantification of total RNA or miRNA and cytokines as predictors of therapeutic effect.

It has been reported that the combination of tangential flow filtration (TFF) technology and ultra-high-speed centrifugation is a convenient and efficient method for enriching EVs. Lorenzini et al. have developed a large-scale cell culture method, in combination with TFF, that can support cell survival and EV production for at least 216 h in both EV-free human platelet lysate (hPL) and fetal calf serum (FCS) environments [131]. This method provides technical support for creating a cell culture environment without contamination from external EVs. Previous studies have indicated that the use of phosphate-buffered saline (PBS) as a dissolving agent can significantly reduce EVs recovery. However, it has recently been found that by using serum albumin and trehalose (PBS-HAT) as an alternative to PBS, EVs products can be stored at -80 °C for long periods of time [132]. In addition, the efficiency and loss of vesicles can be minimized by optimizing centrifugation conditions, including sample pretreatment, adjustments in centrifugation speed and time, and the incorporation of other purification techniques based on downstream requirements.

Currently, there are several approaches to increasing yield. One approach is to expand cell culture, such as by utilizing 3D culture or hollow fiber culture, to involve a larger number of cells in the production of vesicles. Another approach is to enhance the production of vesicles through chemical or physical stimulation of cells or direct disruption of reconstituted EV-like nanostructures [133]. Achieving stable and efficient drug encapsulating is a challenge faced by various drug carriers. Hydrophilic macromolecules present the greatest difficulty for membrane loading, while lipophilic compounds offer a simpler and more efficient drug loading process. When dealing with highly soluble compounds, increasing the drug concentration in solution or encapulating the drug in synthetic nanoparticles before wrapping the cell membrane are viable options. Studies have shown that lipid nanoparticles (LNP) are the optimal carriers for nucleic acid drug delivery. EBPs formed by the fusion of LNPs and EVs can reduce the inherent toxicity of LNPs, enhance the functionalities of EVs or modified EVs, and synergistically improve therapeutic efficacy. Furthermore, EBPs obtained by coating polymeric nanoparticles with cell membranes can overcome the limitations of the membrane structure for drug loading and enable controlled drug release triggered by external stimuli such as heat, light, ultrasound, or specific tumor tissue characteristics such as a low pH or high hyaluronic acid environment.

## Perspectives

In this review, various modification and engineering strategies of EBPs for efficient drug delivery in targeted cancer therapy are introduced. Through biomimetic technology, different cell membranes can be fused to produce hybrid EBPs that maintain the unique characteristics of the originating cells, thereby integrating the functions of various cell types into biomimetic nanoparticles. The coating of nanomaterials with cell membranes, such as erythrocyte-derived vesicles, is typically effective in prolonging blood circulation and reducing systemic clearance. Moreover, EBPs derived from immune cells can regulate and mediate immune responses to tumors, while inheriting the targeting abilities of the original cells to tumor tissues. Tumor cells, on the other hand, can be utilized to extract and produce EBPs due to their innate ability to target their own tumors while also possessing active immunity to the same.

In recent years, combination therapy has emerged as a crucial trend in cancer intervention. Extensive research has demonstrated that EBPs represent a new and promising delivery platform for cancer combination therapy. Therefore, comprehending the various engineering strategies of EBPs is crucial for accelerating their clinical application and reducing obstacles in cancer combination therapy. As compared to traditional EVs, EBPs produced through artificial means offer greater efficiency and simplicity in drug delivery, as well as increased feasibility in combining EBPs with other therapeutic modalities to treat tumors. In this regard, some researchers are exploring the possibility of de novo synthesis to achieve a completely artificial synthesis of EBPs. As artificial products can achieve composition control and safety, they may better meet regulatory requirements and aid mass production. Hybrid EBPs based on cell membrane components may be a potential breakthrough in clinical applications. These hybrid EBPs may exhibit better delivery efficiency than conventional synthetic nanoparticles and greater stability than conventional EVs, thus demonstrating high applicability.

Multiple clinical trials have demonstrated the potential efficacy of EBPs. However, several challenges must be addressed before their successful translation from the laboratory to clinical application. Research efforts must be directed towards a deeper understanding of the biological function of EBPs, scale-up production, the development of standardized purification protocols, and evaluation methods. Moreover, the quantitative determination of the physicochemical characterization of EBPs is essential to ensure drug loading, quality control, and stable storage. In order to achieve these goals, it is vital to develop large-scale bioreactors, standardized and reproducible culture conditions, and production programs. Optimization of extraction techniques to obtain sufficient cell membranes and identification and quantification of by-products from the production process are also necessary. To successfully translate EBPs from laboratory research to clinical application, it is essential to improve the purity and storage life of the final product. Manufacturing processes must be conducted under aseptic conditions to ensure high recovery rates of therapeutic EBPs under storage conditions without excessive damage to the biological content and particulate matter. The future of EBPs holds immense promise. These remarkable nanoparticles, inspired by the natural properties of extracellular vesicles (EVs) derived from cells, have the potential to revolutionize drug delivery and targeted therapy. By mimicking the surface characteristics and cargo-loading capabilities of EVs, EBPs can effectively navigate the complexities of the human body, reaching specific target sites with remarkable precision. The use of EBPs opens up new possibilities in personalized medicine, enabling tailored treatments that harness the body’s own natural processes. Furthermore, these nanoparticles demonstrate enhanced stability, biocompatibility, and prolonged circulation time, further augmenting their therapeutic potential. As research advances and technology evolves, we can eagerly anticipate the continued exploration and development of these innovative nanoparticles, bringing us closer to a future where precise and effective therapeutic interventions become a reality.

## Data Availability

Not applicable.

## Abbreviations

EV: Extracellular vesicle
EBP: Cell-generated EV-biomimetic nanoparticles
ISEV: The International Society for Extracellular Vesicles
TLR: Toll-like receptor
LPS: Lipopolysaccharide
TEV: Tumor cell-derived EV
CRISPR: Clustered regularly scattered short palindromic repeating sequence
Cas9: CRISPR-associated proteins 9
PD1: Programmed cell death protein 1
VSV-G: Vesicular stomatitis virus g-glycoprotein
CXCR4: C-X-C motif chemokine receptor 4
scFv: Single-chain fragment variable
TRAIL: Tumor necrosis factor-related apoptosis-inducing ligand
ICG: Indocyanine green
GM-CSF: Granulocyte–macrophage colony-stimulating factor
PLGA: Poly (lactic-co-glycolic acid)
GPC3: Glypican 3
HER2: Human epidermal growth factor receptor 2
EGF: Epidermal growth factor
DOX: Doxorubicin
5-FU: 5-Fluorouracil
CAR: Chimeric antigen receptor
LAMP2b: Lysosome-associated membrane glycoprotein 2b
DARPin: Designed ankyrin repeat protein
PSMA: Prostate-specific membrane antigen
PSA: Prostate-specific antigen
TNF-α: Tumour necrosis factor alpha
MHC-I: Major histocompatibility class I
PARP: Poly (ADP-ribose) polymerase
EGFR: Epidermal growth factor receptor
BFGF: Basic fibroblast growth factor
PD-L1: Programed-cell death ligand 1
ASO: Antisense oligonucleotide
NP: Nano particle
SPION: Superparamagnetic iron oxide nanoparticles
MRI: Magnetic resonance imaging
DSPE-PEG: 1, 2-Distearoyl-sn-glycero-3-phosphoethanolamine-Poly(ethylene glycol)
MOF: Metal–organic framework
DPPC: 1,2-Dipalmitoyl-sn-glycero-3-phosphocholine
CLT: Celastrol
MSPC: 1-Stearoyl-2-hydroxy-sn-glycero-3-phosphocholine
Tf: Transferrin
TfR: Transferrin receptor
DBCO: Dibenzocyclooctyne
PS: Phosphatidylserine
HA: Hyaluronic acid
PDA: Polydopamine
GFP: Green fluorescent protein
RBC: Red blood cell
PTX: Paclitaxel
FA: Folic acid
OMV: Outer membrane vesicles
MNP: Metal-nanoparticle
NHS: n-hydroxysuccinimde
EDC: 1-Ethyl-3-(3-dimethylaminopropyl)carbodiimide
GBM: Glioblastoma
BBB: Blood–brain barrier
ROS: Reactive oxygen species
SIRP-α: Signal-regulatory protein
SSTR: Somatostatin receptor
AA: Aminoethylanisamide
5-ALA: 5-Aminolevulinic acid
PpIX: Protoporphyrin IX
TAM: Tumor-associated macrophages
NMR: Nuclear magnetic resonance
TPL: Triptolide
CDT: Chemokinetic therapy
Ce6: Chlorin e6
MSC: Mesenchymal stem cell
TNBC: Triple negative breast cancer
ICD: Immunogenic cell death
PTT: Photothermal therapy
TAA: Tumor-associated antigen
PAMAM: Polyamine-amine dendrimers
